# Deregulation of miR‐27a may contribute to canine fibroblast activation after coculture with a mast cell tumour cell line

**DOI:** 10.1002/2211-5463.12831

**Published:** 2020-04-01

**Authors:** Matias Aguilera‐Rojas, Soroush Sharbati, Torsten Stein, Ralf Einspanier

**Affiliations:** ^1^ Institute of Veterinary Biochemistry Department of Veterinary Medicine Freie Universität Berlin Germany

**Keywords:** cancer‐associated fibroblasts, dog, miR‐27a, miRNA, tumour microenvironment

## Abstract

The tumour microenvironment comprises a diverse range of cells, including fibroblasts, immune cells and endothelial cells, along with extracellular matrix. In particular, fibroblasts are of significant interest as these cells are reprogrammed during tumorigenesis to become cancer‐associated fibroblasts (CAFs), which in turn support cancer cell growth. MicroRNAs (miRNAs) have been shown to be involved in this intercellular crosstalk in humans. To assess whether miRNAs are also involved in the activation of fibroblasts in dogs, we cocultured primary canine skin fibroblasts with the canine mast cell tumour cell line C2 directly or with C2‐derived exosomes, and measured differential abundance of selected miRNAs. Expression of the CAF markers alpha‐smooth muscle actin (ACTA2) and stanniocalcin 1 confirmed the activation of our fibroblasts after coculture. We show that fibroblasts displayed significant downregulation of miR‐27a and let‐7 family members. These changes correlated with significant upregulation of predicted target mRNAs. Furthermore, RNA interference knockdown of miR‐27a revealed that cyclin G1 (CCNG1) exhibited negative correlation at the mRNA and protein level, suggesting that CCNG1 is a target of miR‐27a in canine fibroblasts and involved in their activation. Importantly, miR‐27a knockdown itself resulted in fibroblast activation, as demonstrated by the formation of ACTA2 filaments. In addition, interleukin‐6 (IL‐6) was strongly induced in our fibroblasts when cocultured, indicating potential reciprocal signalling. Taken together, our findings are consistent with canine fibroblasts being reprogrammed into CAFs to further support cancer development and that downregulation of miR‐27a may play an important role in the tumour–microenvironment crosstalk.

## Graphical abstract

Fibroblasts are reprogrammed during tumorigenesis to become cancer‐associated fibroblasts (CAFs), which support cancer cell growth. MicroRNAs are involved in this intercellular crosstalk. We show that after coculture of canine fibroblasts with the tumour cell line C2, miR‐27a is downregulated, while expression of CAF markers ACTA2 and stanniocalcin 1 confirmed the activation of fibroblasts. Additionally, RNA interference knockdown of miR‐27a by itself led to fibroblast activation.

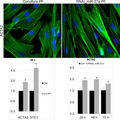

**Abbreviations**
CAFs: Cancer‐associated fibroblasts
CT: Threshold cycle
ECM: Extracellular matrix
IF: Immunofluorescence
miRNA: MicroRNA
PF: Primary fibroblast
RNAi: RNA interference
RT‐qPCR: Reverse transcription–quantitative PCR

Similar to humans, cancer is the leading cause of death in dogs with skin cancer being one of the most common types [1, 2]. Cancer occurs naturally in humans and dogs in similar incidence rates, and since they are companion animals, dogs share the same environment and are exposed to similar risk factors as humans. Moreover, the molecular and clinical resemblances between human and dog cancers in terms of tumour genetics, molecular targets, histological features, response to conventional therapies and age of onset are significant [3, 4]. Hence, the study of oncogenesis in dogs not only is important to further our understanding of the disease within this species, but also is likely to deliver additional valuable information that could be applied in the management of human cancer.

Cancer development implicates complex interactions between genetic and epigenetic modifications, resulting in the ability of cancer cells to escape programmed cell death and grow out of control [5]. As tumour cells proliferate, the surrounding stromal cells, as well as the extracellular matrix (ECM), start to play a dynamic role in the progression of cancer. ECM goes through mechanical and biochemical changes, leading to the remodelling of the local environment that promotes cancer progression. These biological adaptations illustrate the strong and constant interaction between cellular and noncellular components of the tumour microenvironment [6, 7]. The major modulatory role of the ECM in tumour growth has stimulated a significant research interest in fibroblasts, the predominant stromal cell type. Fibroblasts are the cells primarily responsible for the synthesis of ECM, secreting various soluble growth factors and structural proteins, as well as remodelling proteases [8]. Additional factors, including hypoxia, as well as tumour‐ and non‐tumour‐derived cytokines and other signalling molecules, also influence the local tumour microenvironment [9, 10].

Tumour and wound stroma share many similarities such as fibroblast activation, increased production of ECM proteins and enhanced tissue remodelling [11]. When the wound healing process is completed, all these modifications return to their homeostatic state. In contrast, during tumorigenesis, these changes are perpetuated and cancer‐associated fibroblasts (CAFs) remain activated, supporting cancer development [10, 12]. Despite some studies suggesting that most CAFs originate from quiescent local fibroblasts, further investigations have shown that they may also arise from the bone marrow, adipocytes and endothelial or epithelial cells going through mesenchymal transition [8, 13].

Since genetic alterations in CAFs appear only at very low frequency, activation of CAFs seems to be led by epigenetic modifications instead [14]. MicroRNAs (miRNAs) are recognised as one of the major epigenetic gene regulators capable of influencing wide networks of genes at a post‐transcriptional level [15, 16]. miRNAs are short noncoding RNA molecules whose dysregulation has been observed in all types of cancer. They function either as oncomiRs by blocking the expression of tumour suppressors or as anti‐oncomiRs through repression of oncogenes, by means of complementary base pairing within the 3′‐UTRs of their mRNA targets, inhibiting their translation or triggering their decay [17, 18]. Research has shown that pathologic expression of miRNAs favours the development of tumour microenvironment by directly or indirectly influencing interactions between cancer cells and CAFs [9, 19].

Recent investigations focussed on the interaction between tumour and stroma have revealed that cancer cells can reprogramme fibroblasts to become CAFs via miRNAs. In return, CAFs support tumour cell growth, invasion and metastasis, and again, miRNAs appear to be involved in this process [6, 12, 19]. For example, dysregulation of let‐7 family members and miR‐27a, along with several of their target mRNAs, has been found to be involved in regulatory mechanisms of ECM and CAF metabolism [6, 9, 20]. Some of this intercellular crosstalk occurring within the tumour microenvironment is also attributed to exosomes [21]. Exosomes are small membrane vesicles secreted by all types of cells capable of mediating cell‐to‐cell communication by means of exchanging DNA, proteins, mRNAs and miRNAs [22, 23]. For instance, *in vitro* and *in vivo* models for the human disease have shown that exosome‐derived miR‐27a may regulate the transformation of normal fibroblasts into CAFs and also modulate the growth and metastasis of tumour cells [21].

Given the important role that miRNAs play in cancer tumorigenesis, modification of the expression of particular cancer‐associated miRNAs could therefore represent a valuable tool for therapeutics and management of cancer. In this study, we aimed to identify miRNA‐derived mediators of fibroblast activation in dogs by studying differential expression of miRNAs and their predicted mRNA targets in canine fibroblasts after coculture with cancer cells or after exposure to cancer cell‐derived exosomes. We focussed our research on canine orthologues of miRNAs with known deregulation in human and/or canine cancer. Analysis of our data identifies potential regulatory miRNA‐mediated mechanisms and further implies that the canine disease could be a suitable model for human cancer developmental studies.

## Materials and methods

### Cell culture and total RNA isolation

Primary fibroblasts (PFs) were isolated and cultured as previously described [22], and passage number 5 was used for all experiments. C2 cells, a canine mast cell tumour cell line, were kindly provided by P. Dubreuil (Centre de Recherche en Cancérologie de Marseille, Inserm U1068, Marseille, France), after previous consent of the cell line originator, W. Gold (University of California, San Francisco, School of Medicine, CA, USA), and were cultured as described earlier [22].

All cell culture experiments were carried out using three biological replicates and evaluated after 24, 48, 72 and 96 h. Three different groups were examined: PF control group (PF group), coculture of PF and C2 cell group (CC‐PF group) and C2 exosome‐derived group (Exo‐PF). In all three groups, 24 h before the experiments started, 2.2 × 10^5^ PFs were seeded per well in a standard 6‐well culture plate (Sarstedt, Nümbrecht, Germany). At time zero, culture medium was replaced with exosome‐depleted medium, containing RPMI 1640 medium (Biochrom, Berlin, Germany), supplemented with 10% exosome‐depleted FBS (Gibco, Gaithersburg, MD, USA), 100 U·mL^−1^ penicillin/streptomycin (Biochrom), 1 mm·mL^−1^ sodium pyruvate and 2 mm·mL^−1^ glutamine (both from Sigma, St. Louis, MO, USA). In the CC‐PF group, C2 cells were cocultured with PF in a two‐compartment cell culture system, by means of utilising hanging cell culture inserts (0.4 µm PET) (Merck Millicell, Darmstadt, Germany) that created a lower and an upper compartment. PFs were first seeded in the lower compartment, and then, 2.2 × 10^5^ C2 cells were seeded into the insert (upper compartment). For the Exo‐PF group, exosomes were isolated from C2 cell culture media as described earlier [22], using the commercial kit Total Exosome Isolation Reagent – from cell culture media (Invitrogen, Vilnius, Lithuania), after a previous 48‐h incubation period in exosome‐depleted medium. Exosomes from 2.5 mL medium were pelleted (10 000 ***g***, 60 min, 4 °C), resuspended in PBS and added to the culture medium of all wells of the Exo‐PF group.

RNA isolation in PF was carried out using the mirVana miRNA Isolation Kit (Ambion, Darmstadt, Germany) according to the manufacturer's protocol. RNA quality and quantity was validated as described previously [24].

### Selection of miRNAs and mRNAs, primer design and quantification by RT‐qPCR

A total of 20 known *Canis familiaris* miRNAs were selected on the basis that they had previously been reported to play a role in cancer initiation and/or progression in dogs and humans (Table S1). Corresponding mRNA targets were predicted using the online resources RNAhybrid (https://bibiserv.cebitec.uni-bielefeld.de/rnahybrid) [25], TargetScan (v7.2; http://www.targetscan.org) [26] and miRmap (https://mirmap.ezlab.org/app/) [27]. A list of identified targets was loaded on the database for annotation, visualisation and integrated discovery (https://david.ncifcrf.gov/) [28]. Target genes were selected based on their involvement in signalling pathways related to cancer from Kyoto Encyclopedia of Genes and Genomes pathway classification [29] in annotation summary result.

The quantification of miRNAs and mRNAs through Reverse transcription–quantitative PCR (RT‐qPCR) was performed as previously described [24, 30], using measurements in triplicate (miRNA) or duplicate (mRNA) of three biological samples, based on the 2‐ΔΔCT method [31] and following protocols detailed before [32]. miRNA and mRNA primers were designed as reported earlier [30] or using the tool Primer‐BLAST (National Center for Biotechnology Information). miRNA expression was normalised using RNU6‐2 and miR‐326 as reference genes, while HPRT1 and RPS19 were employed for mRNA normalisation. All four genes exhibited stable expression in our system. The entire set of oligonucleotides used in this study is provided in Tables S2 and S3. All oligonucleotides were synthesised by Sigma‐Aldrich (Darmstadt, Germany).

### Transfection of miRNAs

Primary fibroblasts were seeded and cultured until reaching ≥80% confluency in standard 6‐well culture plates. Cells were cultured as described above and transfected with let‐7a and miR‐27a inhibitors (mirVana® miRNA inhibitor, MH10050 and MH11579; Ambion, Austin, TX, USA) and mimics (mirVana® miRNA mimic, MC10050 and MC11579; Ambion), as well as a nontarget (NT) siRNA (D‐001810‐02‐05; Dharmacon, Lafayette, CO, USA) as control and a fluorescent TAMRA‐labelled siRNA (Sigma‐Aldrich) as transfection efficiency control, using a final concentration of 32 nm. TransIT‐TKO (Mirus, Madison, WI, USA) was employed as transfection reagent, following the manufacturer’s protocol. Six different groups were assessed: NT (siRNA), 7aIH (let‐7a inhibitor), 27aIH (miR‐27a inhibitor), 7a/27aIH (let‐7a plus miR‐27a inhibitors), 7a/27aMM (let‐7a plus miR‐27a mimics) and IF (fluorescent siRNA), along with evaluation of three time points: 24, 48 and 72 h. After 24 h of incubation, the medium was removed from all wells, cells were washed with PBS, and fresh medium was added for further culturing. Cell viability was assessed by immunofluorescence (IF) as described below. RT‐qPCR was used to estimate inhibitor and mimic effects post‐transfection.

### Immunofluorescence

Detection of IF in our experiments was conducted as outlined previously [33], with some modifications described below. Sterile 13‐mm diameter coverslips (Sarstedt) were placed inside of a standard 24‐well culture plate (Sarstedt), and then, 0.5 × 10^5^ PFs were seeded per well and cultured in a two‐compartment cell culture system for 96 h. Cells were washed with cold PBS and fixed with 4% Histofix (Carl Roth, Karlsruhe, Germany) at room temperature for 15 min, then washed twice with PBS for 5 min and permeabilised using 0.25% (v/v) Triton X‐100/PBS for 5 or 10 min at room temperature. A blocking step with 1% BSA/PBST [0.1% (v/v) Tween‐100 and 1% (w/v) BSA in PBS] was performed for 1 h at room temperature. Coverslips were taken from the wells and laid on a 40‐µL drop of primary antibody dilution in 1% BSA/PBST for 1 h at room temperature. After washing twice with PBS for 5 min, the secondary antibody was incubated for 1 h at room temperature (40‐µL drop). Cells were washed twice with PBS, and nuclei were counterstained with 1 µg·mL^−1^ 4′,6‐diamidin‐2‐phenylindol (Sigma‐Aldrich) in PBS for 3 min at room temperature. Coverslips were mounted in 50% glycerol in PBS on a glass slide.

To detect IF in transfected groups, 0.25 × 10^5^ PFs were seeded per well in an 8‐well cell culture chamber slide (Sarstedt), cultured and transfected as described above. The IF staining was carried out as in coculture experiments, except from the primary antibody incubation step, which was performed afterwards on the chamber slides.

Primary antibodies were as follows: Anti‐vimentin (1 : 50, #5741; CST, Danvers, MA, USA) rabbit antibody was used as fibroblast marker (Fig. S1); anti‐ACTA2 (1 : 300, AJ1028a; Abgent, San Diego, CA, USA), and anti‐CCNG1 and anti‐JAK2 (both at 1 : 200; orb213680 and orb318917, both from Biorbyt, St. Louis, MO, USA) rabbit antibodies were used for IF detection in PF. The secondary antibody was goat anti‐rabbit IgG DyLight 488 (1 : 400, #35553; Thermo Fisher Scientific, Waltham, MA, USA). Cell viability after transfection was assessed using Calcein acetoxymethyl/Hoechst (Calcein‐AM, Biotium, Hamburg, Germany/Hoechst 33342; Thermo Fisher Scientific, Paisley, UK): PFs were incubated with 0.4 µm Calcein‐AM at 37 °C (5% CO_2_, 30 min) and washed twice with PBS. Then, 5 µg·mL^−1^ Hoechst was added to each well, incubated for 5 min at room temperature and washed twice with PBS.

Images were acquired using a Leica DMI6000B inverted microscope and the Leica las‐x software (Leica, Wetzlar, Germany). For direct comparison, IF images were taken under identical microscope and camera settings.

### Western blot

Protein isolation and detection were performed as described earlier [34] using three biological replicates. Briefly, proteins were separated by SDS/PAGE using a Tris/glycine buffer system and transferred onto a polyvinylidene fluoride membrane via semidry blot. Primary antibodies were as follows: anti‐CCNG1 (1 : 1000, orb213680; Biorbyt) rabbit antibody and anti‐ACTB (1 : 1000, sc‐47778; Santa Cruz Biotechnology, Santa Cruz, CA, USA) mouse antibody. Secondary antibodies were as follows: donkey anti‐rabbit and sheep anti‐mouse horseradish peroxidase‐conjugated (1 : 30 000 and 1 : 40 000; NA934 and NA931, both from GE Healthcare, Buckinghamshire, UK) antibodies. Protein quantification was performed by densitometry using the software bio‐1d (Vilber Lourmat, Marne‐la‐Vallée, France).

### Data analysis

A two‐tailed Student’s *t*‐test was used for comparison between the control group and treated groups (two‐group analyses). Results are expressed as means of triplicate or duplicate measurements (technical replicates) ±SD. A *P*‐value < 0.05 was considered statistically significant.

## Results

### Differential expression of miRNAs and their mRNA targets in activated PF

**Fig. 1:**
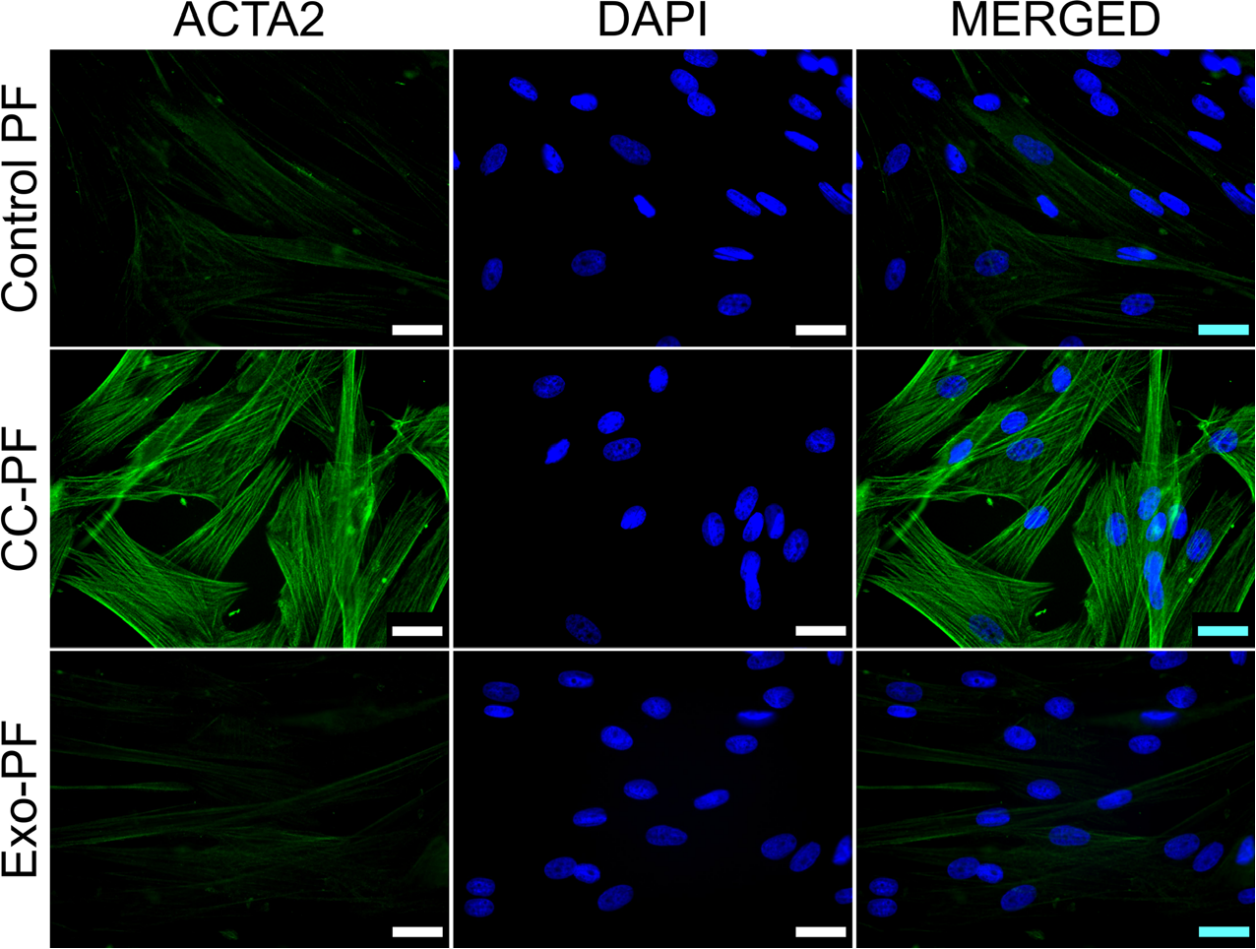
Immunostaining in canine PFs for ACTA2 protein. Images show well‐defined and organised long intracytoplasmic ACTA2 filaments in CC‐PF 96 h after coculture compared with control PF and Exo‐PF groups. IF representative images using at least two biological replicates were taken under identical microscope and camera settings. Scale bars represent 25 μm

We hypothesised that coculture of canine PFs with the canine mast cell tumour cell line C2 (CC‐PF group) would induce an activation of normal fibroblasts and that this activation would be mediated by miRNAs. We also evaluated whether C2‐derived exosomes (Exo‐PF group) could trigger similar effects. We initially assessed this by measuring the expression of the protein alpha‐smooth muscle actin (ACTA2) by IF, the marker most widely used for identifying CAFs [10, 12]. While control PF and Exo‐PF groups showed only weakly positive signals of a diffuse net of filaments, fibroblasts in coculture for 96 h exhibited strong ACTA2 fluorescent signals as well‐defined and organised long intracytoplasmic filaments as would be expected for activated fibroblasts (Fig. 1). Corresponding changes were also observed at mRNA level for ACTA2 and for the CAF‐associated marker stanniocalcin 1 (STC1) [35] (Fig. S2), while asporin (ASPN) and calcium binding protein A4 (S100A4) were not detectable (data not shown). These data confirmed that coculture of the PF with C2 cells did activate our canine fibroblasts.

**Fig. 2:**
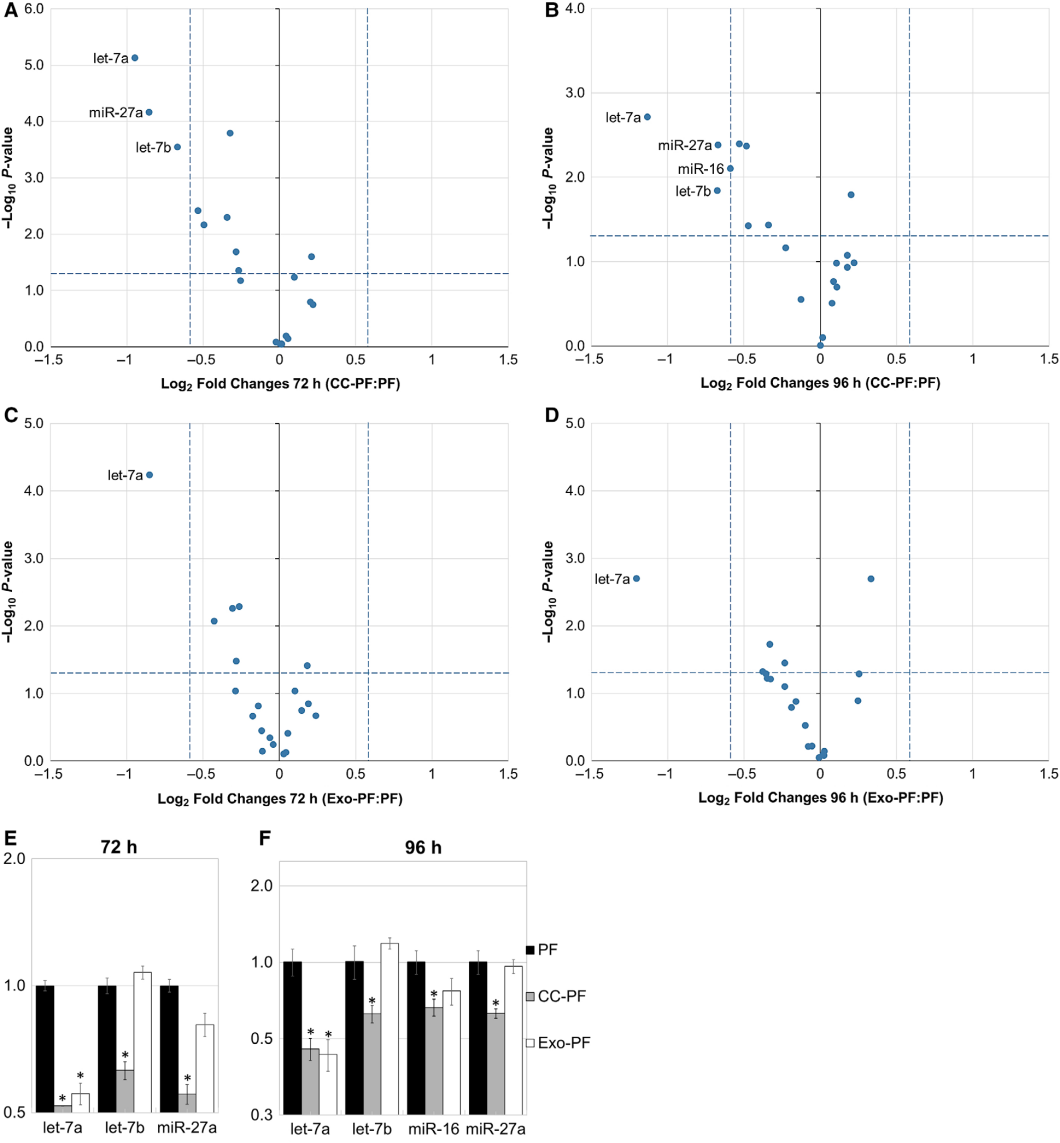
Relative expression of miRNAs in canine PFs compared with PF control group. (A) 72 h and (B) 96 h after coculture with C2 cells: CC‐PF group. (C) 72 h and (D) 96 h after culture with C2‐derived exosomes: Exo‐PF group. Regulated miRNAs in each group and time point (A‐D) are indicated with their names in the plot. Bar charts show only regulated miRNAs after (E) 72 h and (F) 96 h in CC‐PF and Exo‐PF groups. Results were normalised to RNU6‐2 and miR‐326 and analysed using the 2‐ΔΔCT method. Datasets are expressed as means of three biological samples and triplicate measurements ± SD, analysed with a two‐tailed Student’s *t*‐test and transformed into log_2_ vs. –log_10_ *P*‐value for volcano plots (A‐D). Asterisks represent a statistical significance compared with the control group FB (*P < 0.05)

The expression of 20 miRNAs with known dysregulation in human and/or canine cancer (Table S1) was then investigated in C2‐PF coculture experiments to test whether activation of our fibroblasts correlated with a differential regulation of one or more of these miRNAs. While none of these miRNAs were significantly changed during the first 48 h of coculture (Fig. S3), let‐7a, let‐7b and miR‐27a were significantly (*P* < 0.05) reduced more than 1.5‐fold after 72 h (Fig. 2A,E) when compared to the PF control group. This downregulation continued after 96 h of coculture, with additional significant reduction in miR‐16 (Fig. 2B,F).

To test whether similar changes could also be induced by exosome‐derived signals, PFs were cultured in the presence of purified C2‐derived exosomes (Exo‐PF group). During the initial 48 h, no significant changes were observed in the miRNA expression of PF (Fig. S3). However, after 72 h and 96 h let‐7a was again significantly downregulated in the Exo‐PF group (Fig. 2C‐F) when compared to the PF control group, while no changes in miR‐27a expression were observed.

**Fig. 3:**
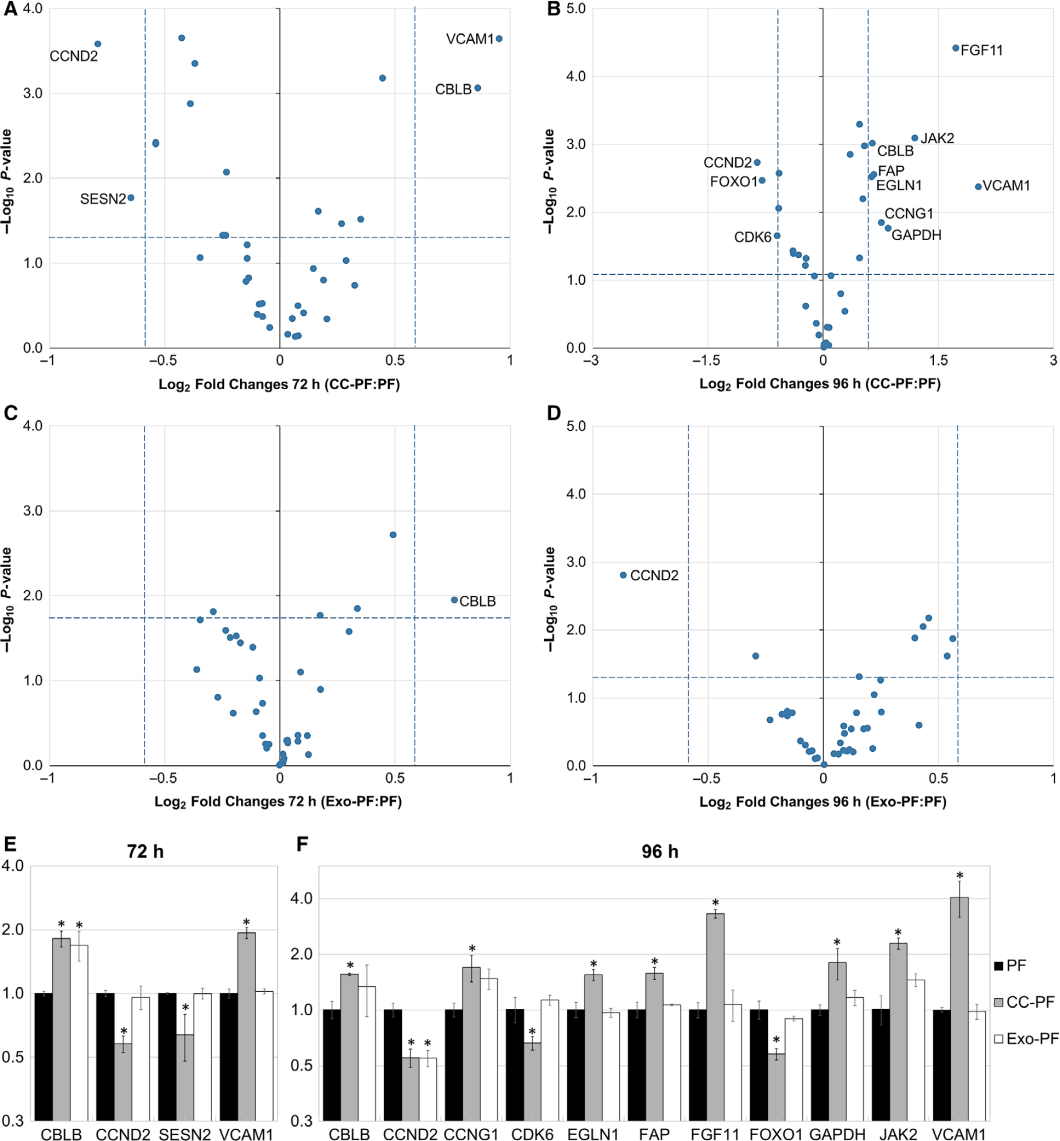
Relative expression of predicted mRNA targets in canine PFs compared with PF control group. (A) 72 h and (B) 96 h after coculture with C2 cells: CC‐PF group. (C) 72 h and (D) 96 h after culture with C2‐derived exosomes: Exo‐PF group. Regulated mRNAs in each group and time point (A–D) are indicated with their names in the plot. Bar charts show only regulated mRNA targets after (E) 72 h and (F) 96 h in CC‐PF and Exo‐PF groups. Results were normalised to HPRT1 and RPS19 and analysed using the 2‐ΔΔCT method. Datasets are expressed as means of three biological samples and duplicate measurements ± SD, analysed with a two‐tailed Student’s *t*‐test and transformed into log_2_ vs. –log_10_ *P*‐value for volcano plots (A‐D). Asterisks represent a statistical significance compared with the control group FB (*P < 0.05)

To evaluate whether the downregulation of the identified miRNAs also led to the upregulation of their predicted common mRNA targets, RT‐qPCRs were performed on PF from both coculture and C2‐derived exosome groups. Again, no significant changes were observed during the first 48 h in either CC‐PF or Exo‐PF groups (Fig. S4). However, after 72 and 96 h the CC‐PF group showed a significant differential regulation of several predicted mRNA targets of more than 1.5‐fold (Fig. 3A‐D) when compared to the PF control group. After 72 h, mRNAs encoding Cbl proto‐oncogene B (CBLB) and vascular cell adhesion molecule 1 (VCAM1) were upregulated, while cyclin D2 (CCND2) and sestrin 2 (SESN2) were downregulated (Fig. 3E). While CBLB and VCAM1 mRNAs were still upregulated after 96 h, significantly increased levels of cyclin G1 (CCNG1), Egl‐9 family hypoxia‐inducible factor 1 (EGLN1), fibroblast‐associated protein (FAP), fibroblast growth factor 11 (FGF11), glyceraldehyde‐3‐phosphate dehydrogenase (GAPDH) and Janus kinase 2 (JAK2) were now additionally observed, while CCND2, cyclin‐dependent kinase 6 (CDK6) and forkhead box O1 (FOXO1) were downregulated (Fig. 3F). mRNA levels of the predicted target interleukin‐6 (IL‐6) were below the detection limit in all time points of the control PF group, but its expression was induced after 72 and 96 h in the CC‐PF group. In contrast, the Exo‐PF group showed very few changes with only CBLB mRNA exhibiting significant upregulation after 72 h (Fig. 3E), while CCND2 was the only downregulated target after 96 h (Fig. 3F). This showed that although coculture with C2‐derived exosomes did lead to a significant reduction in let‐7a in our PF, the exosome‐containing medium was not sufficient to fully replicate the coculture experiment results.

### Selective targeting of mRNAs using miRNA inhibitors and mimics

To test whether the observed reduction in let‐7a and miR‐27a was sufficient to induce activation of PF and to generate the observed changes in mRNA abundance of the predicted target genes, we tried to emulate the effects induced by C2 coculture on PF by means of RNA interference (RNAi)‐mediated knockdown. PFs were transfected either with let‐7a‐ or miR‐27a‐specific inhibitors (7aIH and 27aIH groups), or with their miRNA mimics (7a/27aMM group) as control, along with a NT control siRNA. Parallel transfection with fluorescently labelled control siRNAs indicated that the transfection efficiency was above 90% after 24 h, while cell viability was not visibly affected (Figs S5 and S6).

RT‐qPCRs revealed that cells treated with either miR‐27a inhibitor or mimic showed the expected significant down‐ or upregulation of miR‐27a compared with the NT control. However, while PF transfected with let‐7a mimic showed significantly increased levels of let‐7a, cells transfected with let‐7a inhibitor did not show decreased concentrations of this miRNA (Fig. S7), so that we did not further evaluate these cells. Therefore, predicted mRNA targets, whose expression was upregulated in the CC‐PF group, were only further evaluated after RNAi knockdown of miR‐27a.

**Fig. 4:**
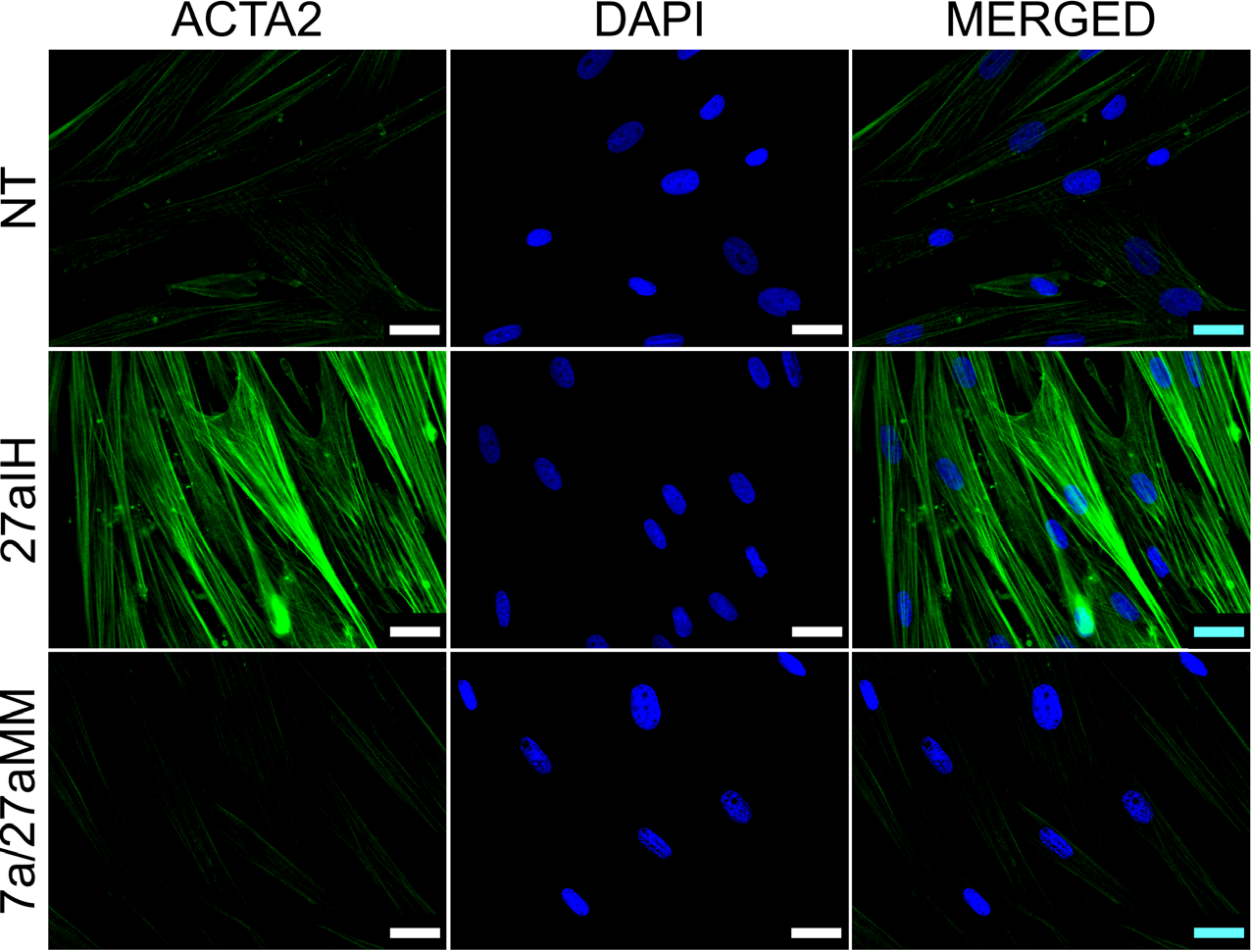
Immunostaining in canine PFs for ACTA2 protein in transfection experiments. Images show the expression of intracytoplasmic ACTA2 filaments 48 h after transfection with miR‐27a inhibitor compared with NT and 7a/27aMM groups. IF representative images using at least two biological replicates were taken under identical microscope and camera settings. Scale bars represent 25 μm

**Fig. 5:**
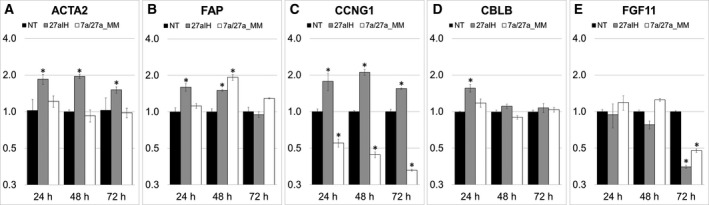
Relative expression of predicted and regulated target mRNAs in canine PFs compared with the NT group. Twenty‐four hours, 48 and 72 h after transfection in 27aIH and 7a/27aMM groups. (A) ACTA2, (B) FAP, (C) CCNG1, (D) CBLB and (E) FGF11. Results were normalised to HPRT1 and RPS19 and analysed using the 2‐ΔΔCT method. Datasets are expressed as means of three biological samples and duplicate measurements ± SD, and analysed with a two‐tailed Student’s *t*‐test. Asterisks represent a statistical significance compared with the control group NT (*P < 0.05)

ACTA2 protein expression was again assessed by IF to measure changes in the activation status of the transfected PF. Forty‐eight hours after transfection with miR‐27a inhibitor, ACTA2 protein was observed as well‐defined and organised long intracytoplasmic filaments consistent with fibroblast activation, while NT and 7a/27aMM groups exhibited only diffuse and weakly positive signals (Fig. 4). These results show that RNAi knockdown of miR‐27a could emulate our coculture results by inducing the expression of ACTA2 at protein level in canine PF. Consistent with our IF results, ACTA2 mRNA levels were significantly increased at 24, 48 and 72 h, and FAP mRNA, at 24 and 48 h (Fig. 5A,B), further confirming the activation of our fibroblasts. CCNG1 also exhibited significant and anticorrelative expression in PF in response to transfection with the miR‐27a inhibitor after 24, 48 and 72 h (Fig. 5C), while CBLB was only significantly upregulated in PF transfected with miR‐27a inhibitor after 24 h (Fig. 5D).

Transfection with the miRNA mimics gave a mixed response, with consistent significant decrease in CCNG1 mRNA (Fig. 5C), while FAP mRNA levels were significantly increased at 48 h (Fig. 5D) and FGF11 displayed reduced mRNA levels 72 h after transfection with inhibitor and mimics (Fig. 5E). Therefore, the inhibitor results indicate that CCNG1, CBLB and FAP, whose predicted miR‐27a binding sites are conserved between dogs and humans (Fig. S8) along with ACTA2, may be the main targets for miR‐27a in our system, while the mimic results were inconclusive.

### Detection of target proteins in PF

Since CCNG1 was the only miR‐27a target exhibiting a consistent and anticorrelative expression in transfected PF in our experiments, CCNG1 protein expression and localisation were further measured in PF by IF and western blot. As transfection of the let‐7a inhibitor did not display significant changes, the 7aIH group was not tested for protein expression.

**Fig. 6:**
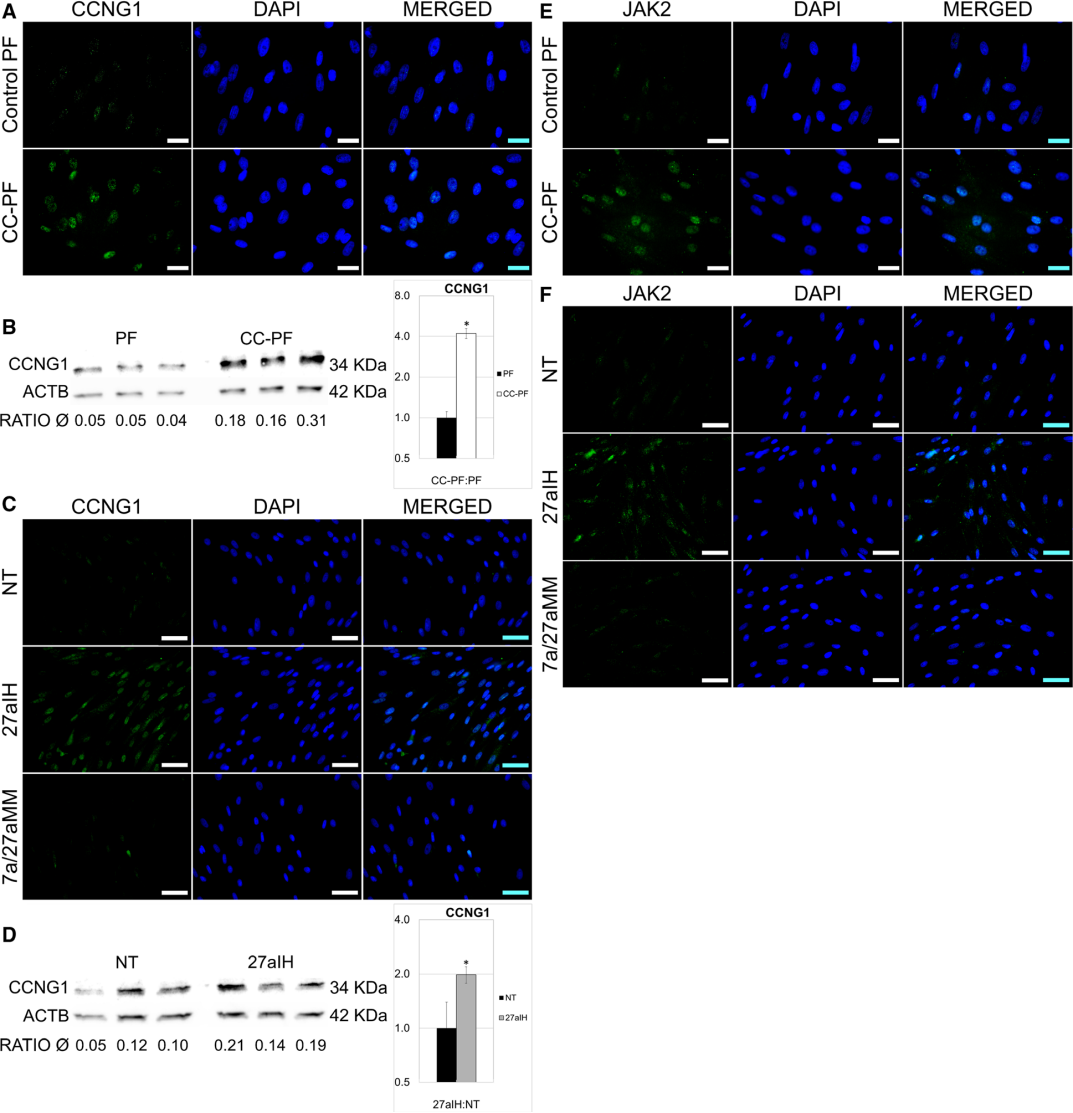
Detection of CCNG1 and JAK2 proteins of in canine PFs. (A) IF staining of CCNG1 in CC‐PF 96 h after coculture compared with control PF. (B) Western blot for CCNG1 in CC‐PF 96 h after coculture compared with the PF control group. (C) IF staining of CCNG1 72 h after transfection with 27aIH (miR‐27a inhibitor) compared with NT (siRNA) and 7a/27aMM (let‐7a plus miR‐27a mimics) groups. (D) Western blot for CCNG1 in PF 72 h after transfection with miR‐27a inhibitor compared with NT. CCNG1 protein bands were quantified in three biological samples by densitometry relative to the respective beta‐actin (ACTB) signals for both blots (B and D). Charts represent means of the biological triplicates ± SD and analysed with a two‐tailed Student’s *t*‐test. Asterisks represent a statistical significance compared with the control group PF (*P < 0.05). (E) IF staining of JAK2 CC‐PF after 96 h compared with control PF. (F) IF staining of JAK2 72 h after transfection with 27aIH compared with NT and 7a/27aMM groups. IF representative images were taken using at least two biological replicates, under identical microscope and camera settings. Scale bars represent 25 μm (A and E) and 50 µm (C and F).

Figure 6A shows that PF cocultured for 96 h with C2 cells showed visibly increased expression of CCNG1 protein when compared to the control group. CC‐PF cells showed clearly detectable signals for CCNG1 in the nucleus, while the fluorescence observed in control PF under the same conditions was substantially lower. Similar results were observed when PFs were evaluated 72 h after transfection with miR‐27a inhibitor. Again, nuclear fluorescence for CCNG1 was higher in the 27aIH group when compared to the staining of the NT and 7a/27aMM groups (Fig. 6C). Western blots confirmed a significant increase in CCNG1 protein expression in CC‐PF and 27aIH groups when compared to their respective controls (Fig. 6B,D). These observations confirmed the upregulation of CCNG1 protein in our fibroblasts through C2 coculture and transfection with miR‐27a inhibitor, and are consistent with our findings at mRNA level (Figs 3F and 5C).

As JAK2 mRNA expression was also upregulated after 96 h in the CC‐PF group (Fig. 3F), we assessed its protein expression by IF as well. Consistent with our RT‐qPCR results, JAK2‐specific nuclear fluorescence was enhanced in C2 coculture PF compared with the control PF group (Fig. 6E). Similar results were observed in PF transfected with the miR‐27a inhibitor. Seventy‐two hours after transfection, PF showed clearly enhanced nuclear fluorescence for JAK2 when compared to the NT and 7a/27aMM groups (Fig. 6F). Our JAK2 protein expression data therefore correlate with JAK2 mRNA levels observed in CC‐PF (Fig. 3F). Interestingly, while transfection with the miR‐27a inhibitor did not affect JAK2 mRNA expression, IF results showed a visible upregulation at protein level in PF, suggesting a post‐transcriptional regulation of JAK2 protein.

## Discussion

The tumour microenvironment is composed of a heterogeneous stroma including immune cells, endothelial cells and fibroblasts, along with the ECM. CAFs are the most abundant cell type in the stroma of solid tumours and the main producers of ECM [8]. Many of the pro‐oncogenic phenomena and interactions taking place in the tumour microenvironment are driven via miRNAs [17, 19]; hence, there is great interest in understanding how the malignant tumour cell–fibroblast crosstalk affects miRNA abundance in the surrounding environment. However, most of these studies have only been performed in human or rodent systems. To test whether the same crosstalk occurs in dogs, we have for the first time used a systematic approach to study miRNA expression in canine PF after coculture with the mast cell tumour cell line C2. For this purpose, we first assessed changes in abundance of selected canine orthologues of miRNAs in canine PF after coculture with C2 cells and with C2‐derived exosomes, respectively. Secondly, we tried to evaluate computationally predicted mRNA targets *in vitro* via RNAi knockdown to indicate potential mechanisms of fibroblast activation.

Our results show that coculture with C2 cells induced downregulation of let‐7 family members and miR‐27a in canine PF. Furthermore, our data indicate that the growth‐promoting cell cycle regulator CCNG1 is a target of miR‐27a in dogs. PF cultured with C2‐derived exosomes also displayed a significant downregulation of let‐7a, though its predicted targets were not affected. These results indicate that exosomes do not appear to play a major role in modifying gene expression of the selected miRNAs in our PF‐C2 cell system.

let‐7a has been mostly described in humans as a tumour suppressor by targeting cancer‐promoting genes in colon [36], prostate [37] and breast cancer [38]. In fibroblasts, downregulation of let‐7a has been found to promote type I collagen expression, while its overexpression reduced fibrosis [20]. In our system, let‐7a was downregulated in PF after coculture with C2 cells, as well as in PF cultured with C2‐derived exosomes. Unfortunately, transfection with a let‐7a inhibitor did not change the abundance of let‐7a nor of its proposed mRNA targets. Thus, potential targets of let‐7a could not be further assessed in this study.

In humans, miR‐27a expression differs from one cancer type to another, acting either as oncomiR or as anti‐oncomiR. It has been reported that in its oncogenic role, miR‐27a modulates the malignant behaviour in osteosarcoma cells [39], promotes proliferation and invasion in lung cancer cells [40], and supports cell survival and angiogenesis in breast cancer [41]. On the other hand, Zhao and colleagues [42] showed that when functioning as anti‐oncomiR, the downregulation of miR‐27a contributed to metastasis in hepatocellular carcinoma. Likewise, it has been found that increased levels of miR‐27a inhibited cell proliferation and enhanced apoptosis in colorectal [43] and lung cancer [44]. Research into miR‐27a in fibroblasts has also yielded contradictory results. Experimental data demonstrated that exosome‐derived miR‐27a produced oxidative stress in human skin fibroblasts and inhibited their migration [23] and that miR‐27a overexpression hindered lung fibrosis [45]. In contrast, one report indicated that miR‐27a induced the reprogramming of fibroblasts into CAFs and also promoted proliferation, motility and metastasis of gastric cancer cells [21]. Our data have now shown that C2 cells can induce the expression of ACTA2 in PF and that this effect is likely to be mediated by means of miR‐27a downregulation. It is known that normal fibroblasts can adapt to *in vitro* culture systems by promoting markers typically associated with fibroblast activation such as ACTA2 [46], which could explain the weak positive signal for ACTA2 in our control groups. Nevertheless, our results showed a marked increase in ACTA2 fluorescent signals and mRNA upregulation in coculture and transfection experiments. Our data therefore support recently published observations that knockdown of miR‐27a induces the expression of ACTA2 and enhances the differentiation of lung fibroblasts into myofibroblasts, and further support fibrosis [45].

Due to CAF heterogeneity, ACTA2 needs to be evaluated together with other CAF markers, including FAP, an important ECM‐modifying enzyme that plays a significant role in matrix remodelling [7, 10], and STC1, a glycoprotein secreted by activated CAFs with a protumorigenic role [35, 47]. Both FAP and STC1 mRNA levels were increased in C2‐cocultured PF, while FAP mRNA upregulation was also detected in miR‐27a knockdown fibroblasts, confirming their activation and further supporting our hypothesis of tumour cell‐derived activation of fibroblasts. However, other proposed CAF markers including ASPN and S100A4 [47] were not induced, confirming the variability between CAFs.

Analysis of the potential miR‐27a targets further identified possible mechanisms of the fibroblast activation in our system. Expression of CCNG1 was enhanced in CC‐PF after 96 h both at mRNA and protein levels, as well as in PF 24, 48 and 72 h after transfection with miR‐27a inhibitor. CCNG1 expression has been associated with growth promotion and cell cycle progression [48]. The oncogenic behaviour of CCNG1 has been well documented, and its overexpression has been detected in several types of cancers [49, 50], yet a cell growth‐inhibitory function of CCNG1 has also been suggested [51]. However, in a study of normal human fibroblasts, transfection with a CCNG1 expression vector induced clonal expansion [52]. Furthermore, luciferase reporter assays confirmed that CCNG1 is a direct target of miR‐27a in human osteosarcoma cells [51]. Our data also exhibited significantly upregulated CBLB mRNA levels in CC‐PF after 72 and 96 h, as well as 24 h after transfection with miR‐27a inhibitor. CBLB is a well‐described oncogene, and its inhibition enhances anticancer immunity [53]. Our data are therefore consistent with a role of miR‐27a as tumour suppressor and that its downregulation leads to an upregulation of the pro‐oncogenic CBLB and CCNG1. Interestingly, a report indicated that miR‐27b, another member of the miR‐27 family, directly targets CBLB [54], though it is not clear whether miR‐27a and miR‐27b can interact with the same mRNA targets or are cell type‐specific.

In addition to these possible fibroblast activation models by C2 cells, our data further revealed a potential mechanism for reciprocal fibroblast–tumour cell communication. Studies have shown a proproliferative effect of IL‐6 on CAFs and have correlated its expression with increased levels of the CAF marker ACTA2 [55, 56]. However, IL‐6 is also an important mediator of a dynamic tumour cell–CAF crosstalk by not only promoting fibroblast activation, but also supporting tumour cell growth in humans [57]. IL‐6 was induced in our PF cocultured with C2 cells, since it was not detectable in control PF or in Exo‐PF cultures. Our data therefore are consistent with results described by Karakasheva and colleagues [57], making it likely that the enhanced IL‐6 expression in our fibroblasts is not only part of a fibroblast activation programme but also a reciprocal signal to the tumour cells to enhance their growth. Interestingly, IL‐6 has also been identified as direct target of let‐7a and other members of the let‐7 family [58], suggesting that downregulation of let‐7a and let‐7b observed in CC‐PF could have influenced the expression of IL‐6. In addition, a positive correlation between IL‐6 and both JAK2 [59] and VCAM1 [60] has been previously shown, thereby supporting the results observed in our canine coculture approach.

Three additional upregulated genes in our coculture system further indicated a possible cellular response of PF to an enhanced metabolic status. Canine PF expressed increased mRNA levels of FGF11 and EGLN1 after 96 h. Both genes have been associated with adaptations to metabolic changes that improve cancer cell survival [61]. In contrast to other FGF family members, FGF11 is an intracellular nonsecreted growth factor and shows promitogenic and procell survival activities [62] and is therefore likely to be involved in the fibroblast activation itself. In addition, GAPDH expression was also increased in our cocultured PF, and although it is frequently employed as a housekeeping gene, GAPDH overexpression in fibroblasts has been shown to correlate with fibrosis and with an altered metabolism adapted to support a rapid cell growth [63]. Despite these genes being significantly upregulated at mRNA level after coculture, our transfection results indicated that they do not appear to be direct targets of miR‐27a in our system. Similarly, CCND2, CDK6 and FOXO1 showed decreased mRNA levels in PF after C2 coculture and are consequently unlikely to be direct targets.

Experiments performed in mice showed that normal mast cells can affect fibroblast growth in coculture and enhance their growth rate [64]. We cannot therefore rule out that the observed gene expression changes are an effect of the mast cell origin of C2 cells and not just of their tumour nature. Unfortunately, no suitable normal canine mast cell line that would have allowed us to distinguish between these two possibilities was available to us. Nevertheless, our results have identified potential mechanisms for how C2 cells may induce activation of primary dermal fibroblast that can now be further evaluated.

In conclusion, our data present the first systematic analysis of cellular crosstalk between a canine mast cell tumour and fibroblasts on a miRNA basis, providing essential data for further functional analyses. Our targeted approach identified distinct miRNAs significantly regulated in canine PF after coculture with the mast cell tumour cell line C2 and suggested potential mechanisms for their activation and reciprocal cell crosstalk. Results generated from our coculture system and other *in vitro* experiments have provided evidence that miR‐27a is able to influence fibroblast protein expression associated with their activation. Therefore, our findings in this canine model are consistent with known human cancer cell interactions, reinforcing the idea that canine PFs are also reprogrammed into CAFs and may support cancer development. Downregulation of miR‐27a could therefore play an important role in shaping the cancer microenvironment by further promoting the expression of its cancer‐related targets in dogs. Our data thus further strengthen the concept of the dog as a suitable cancer model for humans.

## Conflict of interest

The authors declare no conflict of interest.

## Author contributions

MAR wrote the original manuscript, performed experiments and analysed data. SS revised and edited the manuscript, designed study, analysed data and supervised planning and execution. TS revised and edited the manuscript, provided experimental advice and analysed data. RE revised and edited the manuscript, provided advice in study design and supervised planning and execution. All authors have read and approved the final version of the manuscript.

## Supporting information


**Table S1.** Literature overview of the selected 20 miRNAs. Expression patterns and their association with cancer in dogs and humans.

**Table S2.** Primer sequences of selected miRNAs. Genes for normalisation are presented in Italic.

**Table S3.** Primer sequences of selected mRNAs. Genes for normalisation are presented in Italic.

**Fig. S1.** Immunofluorescence staining of vimentin in canine primary fibroblasts. IF representative images represent at least two biological replicates. Scale bars represent 50 μm.

**Fig. S2.** Relative expression of mRNA levels for CAF markers ACTA2 and STC1 in canine primary fibroblasts compared with PF control group. Expression was evaluated in PF, CC‐PF and Exo‐PF groups after (A) 72 h and (B) 96 h. Results were normalised to HPRT1 and RPS19 and analysed using of the 2‐ΔΔCT method. Datasets are expressed as means of three biological samples and duplicate measurements ± SD, analysed with a two‐tailed Student’s *t*‐test. Asterisks represent a statistical significance compared with the control group FB (*P =< 0.05).

**Fig. S3.** Relative expression of miRNAs in canine primary fibroblasts compared with PF control group. (A) 24 h and (B) 48 h after coculture with C2 cells: CC‐PF group. (C) 24 h and (D) 48 h after culture with C2‐derived exosomes: Exo‐PF group. No significantly regulated miRNAs were observed. Results were normalised to RNU6‐2 and miR‐326 and analysed using the 2‐ΔΔCT method. Datasets are expressed as means of three biological samples and triplicate measurements ± SD, analysed with a two‐tailed Student’s *t*‐test and transformed into log_2_ vs. –log_10_ *P*‐value. No statistical significance was found compared with the control group FB (*P* < 0.05).

**Fig. S4.** Relative expression of predicted target mRNAs in canine primary fibroblasts compared with PF control group. (A) 24 h and (B) 48 h after coculture with C2 cells: CC‐PF group. (C) 24 h and (D) 48 h after culture with C2‐derived exosomes: Exo‐PF group. No significantly regulated mRNAs were observed. Results were normalised to HPRT1 and RPS19 and analysed using of the 2^‐^ΔΔCT method. Datasets are expressed as means of three biological samples and duplicate measurements ± SD, analysed with a two‐tailed Student’s *t*‐test and transformed into log_2_ vs. –log_10_ *P*‐value. No statistical significance was found compared with the control group FB (*P* < 0.05).

**Fig. S5.** Representative images of transfection efficiency evaluated using a TAMRA‐labelled siRNA. At the same time, viability of primary fibroblasts transfected with fluorescent siRNA control was also evaluated through Calcein and Hoechst fluorescent staining. IF representative images using at least two biological replicates were taken under identical microscope and camera settings. Scale bars represent 100 μm.

**Fig. S6.** Representative images of cell viability in primary fibroblasts evaluated by using Calcein and Hoechst fluorescent staining. (A) Cellular viability in NT group, (B) cellular viability in 27aIH group and (C) cellular viability in 7a/27aMM group. IF representative images using at least two biological replicates were taken under identical microscope and camera settings. Scale bars represent 100 μm.

**Fig. S7.** Relative expression of transfected miRNAs in primary fibroblasts compared with NT group. (A) 7aIH, (B) 27aIH, (C) 7a/27aIH and (D) 7a/27aMM groups. Results were normalised to RNU6‐2 and miR‐326 and analysed using the 2^‐^ΔΔCT method. Datasets are expressed as means of three biological samples and triplicate measurements ± SD, and analysed with a two‐tailed Student’s *t*‐test. Asterisks represent a statistical significance compared with the control group NT (*=*P* < 0.05).

**Fig. S8.** Canine predicted binding sites in the 3’ UTR region of regulated genes after miR‐27a transfection, compared to human. Predicted interaction of each gene’s target region (top) with miR‐27a (bottom). (A) CCNG1 sequence has in both species 2 miR‐27a binding sites in different positions due to nucleotide repetition. (B) CBLB and (C) FAP sequences have in both species a single miR‐27a binding site. Pairing between genes and miR‐27a was performed using the online resources TargetScan and RNAhybrid.
