# Supplementary material for: Deregulation of miR‐27a may contribute to canine fibroblast activation after coculture with a mast cell tumour cell line
Source: FEBS Open Bio. 2020 Apr 1;10(5):802–16. doi: 10.1002/2211-5463.12831 (PMC7193169; doi:10.1002/2211-5463.12831)
Supplement: Supplementary file 1 — Table S1. Literature overview of the selected 20 miRNAs. Expression patterns and their association with cancer in dogs and humans. [file FEB4-10-802-s009.docx]

**Table S1. Literature overview of the selected 20 miRNAs.** Expression patterns and their association with cancer in dogs and humans.

| Nr | miRNA | | Association with cancer |
| --- | --- | --- | --- |
| 1 | let-7a | Tumour suppressor in human gastric [1], PTC [2], colon [3] and mammary gland cancers [4-6] | |
| 2 | let-7b | Tumour suppressor in human mammary gland cancer (as part of the let-7 family) [5] and in human mucosal melanoma [7]. Inhibited cancer-promoting effects of breast CAFs [8] | |
| 3 | miR-9 | Upregulated in canine and human mammary gland cancer [9]. Promoted cancer metastasis in human mammary gland cancer [10] and progression of canine mast cell tumours [11]. Overexpressed in canine OS cells [12] | |
| 4 | miR-15a | Downregulated in canine and human CLL [13, 14]. Downregulated in canine ductal carcinomas [15]. Downregulated in fibroblasts surrounding prostate tumours [16] | |
| 5 | miR-16 | Downregulated in canine and human CLL [13, 14]. Downregulated in fibroblasts surrounding prostate tumours [16] | |
| 6 | miR-18a | Upregulated in canine [17] and human prostate cancer [18]. Tumour suppressor in human ovarian cancer [19]. Promoted cellular proliferation in human mammary gland cancer [20] | |
| 7 | miR-21 | Upregulated in human and canine B-CLL [13] and mammary gland cancers [9, 15]. Associated with increased resistance of various cancers to drug treatments [21]. Induced transformation of fibroblasts into CAFs [22] | |
| 8 | miR-27a | Promoted proliferation in human OS [23], lung cancer [24] and breast cancer [25]. Tumour suppressor in human HCC [26], CRC [27] and lung cancer [28]. Inhibited migration in human fibroblasts [29] and hindered lung fibrosis [30]. Reprogrammed fibroblasts into CAFs and promoted proliferation of gastric cancer cells [31]. | |
| 9 | miR-34a | Downregulated in canine and human mammary gland cancer [9]. Upregulated in canine bladder TCC [32]. Downregulated in canine and human OS [33]. Downregulated in CAF-derived exosomes in oral squamous cell carcinoma [34] | |
| 10 | miR-122 | Tumour suppressor in human HCC [35] and mammary gland cancer [36] | |
| 11 | miR-124 | Tumour suppressor in human CRC [37], squamous cell carcinoma [38], gastric [39] ovarian [40] and lung cancers [41]. Inhibited transition of normal fibroblasts into CAFs [42] | |
| 12 | miR-141 | Downregulated in canine kidney epithelial cells undergone EMT [43] and in human mammary gland cancer cells [44] and CRC [45]. Upregulated in human prostate cancer [46]. Inhibits transition of normal fibroblasts into CAFs [47] | |
| 13 | miR-145 | Downregulated in canine and human mammary gland cancer [9]. Tumour suppressor in canine and human MM [48, 49]. Downregulated in human prostate cancer [46]. Reverted CAFs to a normal fibroblast phenotype [50] | |
| 14 | miR-146a | Upregulated in human mammary cancers [51, 52] and in canine MM [49] and prostate cancer [17]. Upregulated in CAFs of pancreatic cancer [53] | |
| 15 | miR-146b | Upregulated in canine [9] and in human mammary cancers [51]. Tumour suppressor in human lung cancer [54]. Promoted human bladder cancer invasion [55] | |
| 16 | miR-155 | Upregulated in human and canine B-CLL [13]. Upregulated in several human mammary gland cancers [9, 56]. Tumour suppressor in canine ovarian cancer [57]. Tumour suppressor in human CRC [58]. Upregulated in human HCC [59] and liposarcoma [60]. Induced ovarian fibroblasts conversion into CAFs [61] | |
| 17 | miR-182 | Upregulated in canine mammary gland cancer [9]. Suppressed EMT in human lung cancer [62]. Promoted cellular proliferation and inhibits apoptosis in human CRC [63] | |
| 18 | miR-191 | Upregulated in human prostate cancer [64], mammary gland cancer [65, 66] and T lymphoblastic leukemia/lymphoma [67]. Promoted tumorigenesis of human CRC [68] | |
| 19 | miR-203 | Downregulated in canine and human MM [69]. Tumour suppressor in canine lymphoma [70]. Downregulated in canine hemangiosarcoma [71]. Downregulated in human CRC [72] | |
| 20 | miR-214 | Downregulated in canine mammary gland cancer [9]. Tumour suppressor in canine hemangiosarcoma [73]. Promoted tumour growth in human OS [74]. Upregulated in canine OS [75]. Inhibited the tumour-promoting effect of CAFs in gastric cancer [76] | |

PTC: papillary thyroid carcinoma. OS: Osteosarcoma. CLL: chronic lymphocytic leukaemia. B-CLL: B-cell chronic lymphocytic leukaemia. CAFs: cancer-associated fibroblasts. HCC: Hepatocellular carcinoma. CRC: colorectal cancer. EMT: Epithelial to mesenchymal transition. MM: malignant melanoma. TCC: transitional cell carcinoma.

**References for Table S1**

1. Tang, R., Yang, C., Ma, X., Wang, Y., Luo, D., Huang, C., Xu, Z., Liu, P. & Yang, L. (2016) MiR-let-7a inhibits cell proliferation, migration, and invasion by down-regulating PKM2 in gastric cancer, *Oncotarget.* **7**, 5972.

2. Zhou, B., Shan, H., Su, Y., Xia, K., Zou, R. & Shao, Q. (2017) Let-7a inhibits migration, invasion and tumor growth by targeting AKT2 in papillary thyroid carcinoma, *Oncotarget.* **8**, 69746.

3. Li, B., Chen, P., Chang, Y., Qi, J., Fu, H. & Guo, H. (2016) Let-7a inhibits tumor cell growth and metastasis by directly targeting RTKN in human colon cancer, *Biochemical and biophysical research communications.* **478**, 739-745.

4. Kim, S.-J., Shin, J.-Y., Lee, K.-D., Bae, Y.-K., Sung, K. W., Nam, S. J. & Chun, K.-H. (2012) MicroRNA let-7a suppresses breast cancer cell migration and invasion through downregulation of CC chemokine receptor type 7, *Breast Cancer Research.* **14**, R14.

5. Thammaiah, C. K. & Jayaram, S. (2016) Role of let-7 family microRNA in breast cancer, *Non-coding RNA research.* **1**, 77-82.

6. Liu, C., Chen, Z., Fang, M. & Qiao, Y. (2019) MicroRNA let-7a inhibits proliferation of breast cancer cell by downregulating USP32 expression, *TRANSLATIONAL CANCER RESEARCH.* **8**, 1763-+.

7. Tang, H., Ma, M., Dai, J., Cui, C., Si, L., Sheng, X., Chi, Z., Xu, L., Yu, S. & Xu, T. (2019) miR-let-7b and miR-let-7c suppress tumourigenesis of human mucosal melanoma and enhance the sensitivity to chemotherapy, *Journal of Experimental & Clinical Cancer Research.* **38**, 212.

8. Al-Harbi, B., Hendrayani, S.-F., Silva, G. & Aboussekhra, A. (2018) Let-7b inhibits cancer-promoting effects of breast cancer-associated fibroblasts through IL-8 repression, *Oncotarget.* **9**, 17825.

9. Lutful Kabir, F. M., DeInnocentes, P. & Bird, R. C. (2015) Altered microRNA expression profiles and regulation of INK4A/CDKN2A tumor suppressor genes in canine breast cancer models, *Journal of cellular biochemistry.* **116**, 2956-2969.

10. Ma, L., Young, J., Prabhala, H., Pan, E., Mestdagh, P., Muth, D., Teruya-Feldstein, J., Reinhardt, F., Onder, T. T. & Valastyan, S. (2010) miR-9, a MYC/MYCN-activated microRNA, regulates E-cadherin and cancer metastasis, *Nature cell biology.* **12**, 247.

11. Fenger, J. M., Bear, M. D., Volinia, S., Lin, T.-Y., Harrington, B. K., London, C. A. & Kisseberth, W. C. (2014) Overexpression of miR-9 in mast cells is associated with invasive behavior and spontaneous metastasis, *BMC cancer.* **14**, 84.

12. Fenger, J. M., Roberts, R. D., Iwenofu, O. H., Bear, M. D., Zhang, X., Couto, J. I., Modiano, J. F., Kisseberth, W. C. & London, C. A. (2016) MiR-9 is overexpressed in spontaneous canine osteosarcoma and promotes a metastatic phenotype including invasion and migration in osteoblasts and osteosarcoma cell lines, *BMC cancer.* **16**, 784.

13. Wagner, S., Willenbrock, S., Nolte, I. & Murua Escobar, H. (2013) Comparison of non-coding RNAs in human and canine cancer, *Frontiers in genetics.* **4**, 46.

14. Calin, G. A., Dumitru, C. D., Shimizu, M., Bichi, R., Zupo, S., Noch, E., Aldler, H., Rattan, S., Keating, M. & Rai, K. (2002) Frequent deletions and down-regulation of micro-RNA genes miR15 and miR16 at 13q14 in chronic lymphocytic leukemia, *Proceedings of the National Academy of Sciences.* **99**, 15524-15529.

15. Boggs, R. M., Wright, Z. M., Stickney, M. J., Porter, W. W. & Murphy, K. E. (2008) MicroRNA expression in canine mammary cancer, *Mammalian Genome.* **19**, 561-569.

16. Musumeci, M., Coppola, V., Addario, A., Patrizii, M., Maugeri-Sacca, M., Memeo, L., Colarossi, C., Francescangeli, F., Biffoni, M. & Collura, D. (2011) Control of tumor and microenvironment cross-talk by miR-15a and miR-16 in prostate cancer, *Oncogene.* **30**, 4231.

17. Kobayashi, M., Saito, A., Tanaka, Y., Michishita, M., Kobayashi, M., Irimajiri, M., Kaneda, T., Ochiai, K., Bonkobara, M. & Takahashi, K. (2017) MicroRNA expression profiling in canine prostate cancer, *Journal of Veterinary Medical Science*, 16-0279.

18. Hsu, T., Hsu, C., Lee, K., Lin, J., Chen, C., Chang, K.-C., Su, C.-Y., Hsiao, M. & Lu, P.-J. (2014) MicroRNA-18a is elevated in prostate cancer and promotes tumorigenesis through suppressing STK4 in vitro and in vivo, *Oncogenesis.* **3**, e99.

19. Liu, P., Qi, X., Bian, C., Yang, F., Lin, X., Zhou, S., Xie, C., Zhao, X. & Yi, T. (2017) MicroRNA-18a inhibits ovarian cancer growth via directly targeting TRIAP1 and IPMK, *Oncology letters.* **13**, 4039-4046.

20. Janssen, E. A., Slewa, A., Gudlaugsson, E., Jonsdottir, K., Skaland, I., Søiland, H. & Baak, J. P. (2010) Biologic profiling of lymph node negative breast cancers by means of microRNA expression, *Modern Pathology.* **23**, 1567.

21. Hong, L., Han, Y., Zhang, Y., Zhang, H., Zhao, Q., Wu, K. & Fan, D. (2013) MicroRNA-21: a therapeutic target for reversing drug resistance in cancer, *Expert opinion on therapeutic targets.* **17**, 1073-1080.

22. Kunita, A., Morita, S., Irisa, T. U., Goto, A., Niki, T., Takai, D., Nakajima, J. & Fukayama, M. (2018) MicroRNA-21 in cancer-associated fibroblasts supports lung adenocarcinoma progression, *Scientific reports.* **8**, 8838.

23. Salah, Z., Arafeh, R., Maximov, V., Galasso, M., Khawaled, S., Abou-Sharieha, S., Volinia, S., Jones, K. B., Croce, C. M. & Aqeilan, R. I. (2015) miR-27a and miR-27a* contribute to metastatic properties of osteosarcoma cells, *Oncotarget.* **6**, 4920.

24. Chae, D. K., Ban, E., Yoo, Y. S., Kim, E. E., Baik, J. H. & Song, E. J. (2017) MIR‐27a regulates the TGF‐β signaling pathway by targeting SMAD2 and SMAD4 in lung cancer, *Molecular carcinogenesis.* **56**, 1992-1998.

25. Mertens-Talcott, S. U., Chintharlapalli, S., Li, X. & Safe, S. (2007) The oncogenic microRNA-27a targets genes that regulate specificity protein transcription factors and the G2-M checkpoint in MDA-MB-231 breast cancer cells, *Cancer research.* **67**, 11001-11011.

26. Zhao, N., Sun, H., Sun, B., Zhu, D., Zhao, X., Wang, Y., Gu, Q., Dong, X., Liu, F. & Zhang, Y. (2016) miR-27a-3p suppresses tumor metastasis and VM by down-regulating VE-cadherin expression and inhibiting EMT: an essential role for Twist-1 in HCC, *Scientific reports.* **6**, 23091.

27. Bao, Y., Chen, Z., Guo, Y., Feng, Y., Li, Z., Han, W., Wang, J., Zhao, W., Jiao, Y. & Li, K. (2014) Tumor suppressor microRNA-27a in colorectal carcinogenesis and progression by targeting SGPP1 and Smad2, *PloS one.* **9**, e105991.

28. Yan, X., Yu, H., Liu, Y., Hou, J., Yang, Q. & Zhao, Y. (2019) miR-27a-3p Functions as a Tumor Suppressor and Regulates Non-Small Cell Lung Cancer Cell Proliferation via Targeting HOXB8, *Technology in cancer research & treatment.* **18**, 1533033819861971.

29. Tan, W., Zhang, Y., Li, M., Zhu, X., Yang, X., Wang, J., Zhang, S., Zhu, W., Cao, J. & Yang, H. (2019) miR-27a-containing Exosomes Secreted by Irradiated Skin Keratinocytes Delayed the Migration of Unirradiated Skin Fibroblasts, *International Journal of Biological Sciences.* **15**, 2240-2255.

30. Cui, H., Banerjee, S., Xie, N., Ge, J., Liu, R.-M., Matalon, S., Thannickal, V. J. & Liu, G. (2016) MicroRNA-27a-3p is a negative regulator of lung fibrosis by targeting myofibroblast differentiation, *American journal of respiratory cell and molecular biology.* **54**, 843-852.

31. Wang, J., Guan, X., Zhang, Y., Ge, S., Zhang, L., Li, H., Wang, X., Liu, R., Ning, T. & Deng, T. (2018) Exosomal miR-27a derived from gastric cancer cells regulates the transformation of fibroblasts into cancer-associated fibroblasts, *Cellular Physiology and Biochemistry.* **49**, 869-883.

32. Vinall, R. L., Kent, M. S. & deVere White, R. W. (2012) Expression of microRNAs in urinary bladder samples obtained from dogs with grossly normal bladders, inflammatory bladder disease, or transitional cell carcinoma, *American journal of veterinary research.* **73**, 1626-1633.

33. Lopez, C. M., Peter, Y. Y., Zhang, X., Yilmaz, A. S., London, C. A. & Fenger, J. M. (2018) MiR-34a regulates the invasive capacity of canine osteosarcoma cell lines, *PloS one.* **13**, e0190086.

34. Li, Y.-y., Tao, Y.-w., Gao, S., Li, P., Zheng, J.-m., Zhang, S.-e., Liang, J. & Zhang, Y. (2018) Cancer-associated fibroblasts contribute to oral cancer cells proliferation and metastasis via exosome-mediated paracrine miR-34a-5p, *EBioMedicine.* **36**, 209-220.

35. Zeisel, M., Pfeffer, S. & Baumert, T. (2012) miR-122 acts as a tumor suppressor in hepatocarcinogenesis in vivo.

36. Wang, B., Wang, H. & Yang, Z. (2012) MiR-122 inhibits cell proliferation and tumorigenesis of breast cancer by targeting IGF1R, *PloS one.* **7**, e47053.

37. Zhang, J., Lu, Y., Yue, X., Li, H., Luo, X., Wang, Y., Wang, K. & Wan, J. (2013) MiR-124 suppresses growth of human colorectal cancer by inhibiting STAT3, *PloS one.* **8**, e70300.

38. Zhao, Y., Ling, Z., Hao, Y., Pang, X., Han, X., Califano, J. A., Shan, L. & Gu, X. (2017) MiR-124 acts as a tumor suppressor by inhibiting the expression of sphingosine kinase 1 and its downstream signaling in head and neck squamous cell carcinoma, *Oncotarget.* **8**, 25005.

39. Liu, F., Hu, H., Zhao, J., Zhang, Z., Ai, X., Tang, L. & Xie, L. (2018) miR‑124‑3p acts as a potential marker and suppresses tumor growth in gastric cancer, *Biomedical reports.* **9**, 147-155.

40. Yuan, L., Li, S., Zhou, Q., Wang, D., Zou, D., Shu, J. & Huang, Y. (2017) MiR‑124 inhibits invasion and induces apoptosis of ovarian cancer cells by targeting programmed cell death 6, *Oncology letters.* **14**, 7311-7317.

41. Wang, M., Meng, B., Liu, Y., Yu, J. & Chen, Q. (2017) MiR-124 inhibits growth and enhances radiation-induced apoptosis in non-small cell lung cancer by inhibiting STAT3, *Cellular Physiology and Biochemistry.* **44**.

42. Zhang, Y., Cai, H., Chen, S., Sun, D., Zhang, D. & He, Y. (2019) Exosomal transfer of miR‐124 inhibits normal fibroblasts to cancer‐associated fibroblasts transition by targeting sphingosine kinase 1 in ovarian cancer, *Journal of cellular biochemistry*.

43. Gregory, P. A., Bert, A. G., Paterson, E. L., Barry, S. C., Tsykin, A., Farshid, G., Vadas, M. A., Khew-Goodall, Y. & Goodall, G. J. (2008) The miR-200 family and miR-205 regulate epithelial to mesenchymal transition by targeting ZEB1 and SIP1, *Nature cell biology.* **10**, 593.

44. Li, P., Xu, T., Zhou, X., Liao, L., Pang, G., Luo, W., Han, L., Zhang, J., Luo, X. & Xie, X. (2017) Downregulation of miRNA‐141 in breast cancer cells is associated with cell migration and invasion: involvement of ANP32E targeting, *Cancer medicine.* **6**, 662-672.

45. Liang, Z., Li, X., Liu, S., Li, C., Wang, X. & Xing, J. (2019) MiR-141–3p inhibits cell proliferation, migration and invasion by targeting TRAF5 in colorectal cancer, *Biochemical and biophysical research communications.* **514**, 699-705.

46. Richardsen, E., Andersen, S., Melbø-Jørgensen, C., Rakaee, M., Ness, N., Al-Saad, S., Nordby, Y., Pedersen, M. I., Dønnem, T. & Bremnes, R. M. (2019) MicroRNA 141 is associated to outcome and aggressive tumor characteristics in prostate cancer, *Scientific reports.* **9**, 386.

47. Zhou, Y., Zhong, J.-H., Gong, F.-S. & Xiao, J. (2019) MiR-141-3p suppresses gastric cancer induced transition of normal fibroblast and BMSC to cancer-associated fibroblasts via targeting STAT4, *Experimental and molecular pathology.* **107**, 85-94.

48. Noguchi, S., Mori, T., Hoshino, Y., Yamada, N., Nakagawa, T., Sasaki, N., Akao, Y. & Maruo, K. (2011) Comparative study of anti-oncogenic microRNA-145 in canine and human malignant melanoma, *Journal of Veterinary Medical Science*, 1108090601-1108090601.

49. Zamarian, V., Catozzi, C., Ressel, L., Finotello, R., Ceciliani, F., Vilafranca, M., Altimira, J. & Lecchi, C. (2019) MicroRNA Expression in Formalin-Fixed, Paraffin-Embedded Samples of Canine Cutaneous and Oral Melanoma by RT-qPCR, *Veterinary pathology.* **56**, 848-855.

50. Melling, G. E., Flannery, S. E., Abidin, S. A., Clemmens, H., Prajapati, P., Hinsley, E. E., Hunt, S., Catto, J. W., Coletta, R. D. & Mellone, M. (2018) A miRNA-145/TGF-β1 negative feedback loop regulates the cancer-associated fibroblast phenotype, *Carcinogenesis.* **39**, 798-807.

51. Garcia, A. I., Buisson, M., Bertrand, P., Rimokh, R., Rouleau, E., Lopez, B. S., Lidereau, R., Mikaélian, I. & Mazoyer, S. (2011) Down‐regulation of BRCA1 expression by miR‐146a and miR‐146b‐5p in triple negative sporadic breast cancers, *EMBO molecular medicine.* **3**, 279-290.

52. M’hamed, I. F., Privat, M., Ponelle, F., Penault-Llorca, F., Kenani, A. & Bignon, Y.-J. (2015) Identification of miR-10b, miR-26a, miR-146a and miR-153 as potential triple-negative breast cancer biomarkers, *Cellular Oncology.* **38**, 433-442.

53. Richards, K. E., Zeleniak, A. E., Fishel, M. L., Wu, J., Littlepage, L. E. & Hill, R. (2017) Cancer-associated fibroblast exosomes regulate survival and proliferation of pancreatic cancer cells, *Oncogene.* **36**, 1770.

54. Li, Y., Zhang, H., Dong, Y., Fan, Y., Li, Y. & Zhao, C. (2017) MiR-146b-5p functions as a suppressor miRNA and prognosis predictor in non-small cell lung cancer, *Journal of Cancer.* **8**, 1704.

55. Zhu, J., Xu, C., Ruan, L., Wu, J., Li, Y. & Zhang, X. (2019) MicroRNA-146b Overexpression Promotes Human Bladder Cancer Invasion via Enhancing ETS2-Mediated mmp2 mRNA Transcription, *Molecular Therapy-Nucleic Acids.* **16**, 531-542.

56. Mattiske, S., Suetani, R. J., Neilsen, P. M. & Callen, D. F. (2012) The oncogenic role of miR-155 in breast cancer, *Cancer Epidemiology and Prevention Biomarkers.* **21**, 1236-1243.

57. Qin, W., Ren, Q., Liu, T., Huang, Y. & Wang, J. (2013) MicroRNA‐155 is a novel suppressor of ovarian cancer‐initiating cells that targets CLDN1, *FEBS letters.* **587**, 1434-1439.

58. Liu, J., Chen, Z., Xiang, J. & Gu, X. (2018) MicroRNA-155 acts as a tumor suppressor in colorectal cancer by targeting CTHRC1 in vitro, *Oncology letters.* **15**, 5561-5568.

59. Ning, S., Liu, H., Gao, B., Wei, W., Yang, A., Li, J. & Zhang, L. (2019) miR‑155, miR‑96 and miR‑99a as potential diagnostic and prognostic tools for the clinical management of hepatocellular carcinoma, *Oncology letters.* **18**, 3381-3387.

60. Kapodistrias, N., Mavridis, K., Batistatou, A., Gogou, P., Karavasilis, V., Sainis, I., Briasoulis, E. & Scorilas, A. (2017) Assessing the clinical value of microRNAs in formalin-fixed paraffin-embedded liposarcoma tissues: Overexpressed miR-155 is an indicator of poor prognosis, *Oncotarget.* **8**, 6896.

61. Mitra, A. K., Zillhardt, M., Hua, Y., Tiwari, P., Murmann, A. E., Peter, M. E. & Lengyel, E. (2012) MicroRNAs reprogram normal fibroblasts into cancer-associated fibroblasts in ovarian cancer, *Cancer discovery.* **2**, 1100-1108.

62. Li, Y., Zhang, H., Li, Y., Zhao, C., Fan, Y., Liu, J., Li, X., Liu, H. & Chen, J. (2018) MiR‐182 inhibits the epithelial to mesenchymal transition and metastasis of lung cancer cells by targeting the Met gene, *Molecular carcinogenesis.* **57**, 125-136.

63. Perilli, L., Tessarollo, S., Albertoni, L., Curtarello, M., Pastò, A., Brunetti, E., Fassan, M., Rugge, M., Indraccolo, S. & Amadori, A. (2019) Silencing of miR-182 is associated with modulation of tumorigenesis through apoptosis induction in an experimental model of colorectal cancer, *BMC cancer.* **19**, 821.

64. Wang, X., Shi, Z., Liu, X., Su, Y., Li, W., Dong, H., Zhao, L., Li, M., Wang, Y. & Jin, X. (2018) Upregulation of miR-191 promotes cell growth and invasion via targeting TIMP3 in prostate cancer, *JBUON.* **23**, 444-52.

65. Nagpal, N., Ahmad, H. M., Molparia, B. & Kulshreshtha, R. (2013) MicroRNA-191, an estrogen-responsive microRNA, functions as an oncogenic regulator in human breast cancer, *Carcinogenesis.* **34**, 1889-1899.

66. Anwar, S. L., Wulaningsih, W. & Watkins, J. (2017) Profile of the breast cancer susceptibility marker rs4245739 identifies a role for miRNAs, *Cancer biology & medicine.* **14**, 387.

67. Zhang, J., Yang, X., Li, M., Huang, X., Liu, C. & Gao, Z. (2016) Expression of microRNA-191 in T lymphoblastic leukemia/lymphoma and its underlying mechanism, *Zhonghua xue ye xue za zhi= Zhonghua xueyexue zazhi.* **37**, 273-277.

68. Zhang, X.-F., Li, K.-k., Gao, L., Li, S.-Z., Chen, K., Zhang, J.-B., Wang, D., Tu, R.-F., Zhang, J.-X. & Tao, K.-X. (2015) miR-191 promotes tumorigenesis of human colorectal cancer through targeting C/EBPβ, *Oncotarget.* **6**, 4144.

69. Noguchi, S., Mori, T., Hoshino, Y., Yamada, N., Maruo, K. & Akao, Y. (2013) MicroRNAs as tumour suppressors in canine and human melanoma cells and as a prognostic factor in canine melanomas, *Veterinary and comparative oncology.* **11**, 113-123.

70. Uhl, E., Krimer, P., Schliekelman, P., Tompkins, S. M. & Suter, S. (2011) Identification of altered MicroRNA expression in canine lymphoid cell lines and cases of B‐and T‐Cell lymphomas, *Genes, Chromosomes and Cancer.* **50**, 950-967.

71. Grimes, J. A., Prasad, N., Levy, S., Cattley, R., Lindley, S., Boothe, H. W., Henderson, R. A. & Smith, B. F. (2016) A comparison of microRNA expression profiles from splenic hemangiosarcoma, splenic nodular hyperplasia, and normal spleens of dogs, *BMC veterinary research.* **12**, 272.

72. Deng, B., Wang, B., Fang, J., Zhu, X., Cao, Z., Lin, Q., Zhou, L. & Sun, X. (2016) MiRNA-203 suppresses cell proliferation, migration and invasion in colorectal cancer via targeting of EIF5A2, *Scientific reports.* **6**, 28301.

73. Heishima, K., Mori, T., Sakai, H., Sugito, N., Murakami, M., Yamada, N., Akao, Y. & Maruo, K. (2015) MicroRNA-214 promotes apoptosis in canine hemangiosarcoma by targeting the COP1-p53 axis, *PLoS One.* **10**, e0137361.

74. Liu, C. J., Yu, K. L., Liu, G. L. & Tian, D. H. (2015) miR‑214 promotes osteosarcoma tumor growth and metastasis by decreasing the expression of PTEN, *Molecular medicine reports.* **12**, 6261-6266.

75. Heishima, K., Ichikawa, Y., Yoshida, K., Iwasaki, R., Sakai, H., Nakagawa, T., Tanaka, Y., Hoshino, Y., Okamura, Y. & Murakami, M. (2017) Circulating microRNA-214 and-126 as potential biomarkers for canine neoplastic disease, *Scientific reports.* **7**, 2301.

76. Wang, R., Sun, Y., Yu, W., Yan, Y., Qiao, M., Jiang, R., Guan, W. & Wang, L. (2019) Downregulation of miRNA-214 in cancer-associated fibroblasts contributes to migration and invasion of gastric cancer cells through targeting FGF9 and inducing EMT, *Journal of Experimental & Clinical Cancer Research.* **38**, 20.
