# Supplementary material for: Deregulation of miR‐27a may contribute to canine fibroblast activation after coculture with a mast cell tumour cell line
Source: FEBS Open Bio. 2020 Apr 1;10(5):802–16. doi: 10.1002/2211-5463.12831 (PMC7193169; doi:10.1002/2211-5463.12831)
Supplement: Supplementary file 2 — Table S2. Primer sequences of selected miRNAs. Genes for normalisation are presented in Italic. [file FEB4-10-802-s010.docx]

**Table S2. Primer sequences of selected miRNAs.** Genes for normalisation are presented in Italic.

| Nr | miRNA | RT6-miRNA (5'->3') | miRNA-rev (5'->3') |
| --- | --- | --- | --- |
| 1 | let-7a | TGTCAGGCAACCGTATTCACCGTGAGTGGTAACTAT | CGTCAGATGTCCGAGTAGAGGGGGAACGGCGTGAGGTAGTAGGTTGTATA |
| 2 | let-7b | TGTCAGGCAACCGTATTCACCGTGAGTGGTAACCAC | CGTCAGATGTCCGAGTAGAGGGGGAACGGCGTGAGGTAGTAGGTTGTGTG |
| 3 | miR-9 | TGTCAGGCAACCGTATTCACCGTGAGTGGTTCATAC | CGTCAGATGTCCGAGTAGAGGGGGAACGGCGTTCTTTGGTTATCTAGCTGTA |
| 4 | miR-15a | TGTCAGGCAACCGTATTCACCGTGAGTGGTACAAAC | CGTCAGATGTCCGAGTAGAGGGGGAACGGCGTAGCAGCACATAATGGTT |
| 5 | miR-16 | TGTCAGGCAACCGTATTCACCGTGAGTGGTCGCCAA | CGTCAGATGTCCGAGTAGAGGGGGAACGGCGTAGCAGCACGTAAATA |
| 6 | miR-18a | TGTCAGGCAACCGTATTCACCGTGAGTGGTTATCTG | CGTCAGATGTCCGAGTAGAGGGGGAACGGCGTTAAGGTGCATCTAGTGCAG |
| 7 | miR-21 | TGTCAGGCAACCGTATTCACCGTGAGTGGTTCAACA | CGTCAGATGTCCGAGTAGAGGGGGAACGGCGTAGCTTATCAGACTGA |
| 8 | miR-27a | TGTCAGGCAACCGTATTCACCGTGAGTGGTGCGGAA | CGTCAGATGTCCGAGTAGAGGGGGAACGGCGTTCACAGTGGCTAAG |
| 9 | miR-34 | TGTCAGGCAACCGTATTCACCGTGAGTGGTACAACC | CGTCAGATGTCCGAGTAGAGGGGGAACGGCGTGGCAGTGTCTTAGCTGGT |
| 10 | miR-122 | TGTCAGGCAACCGTATTCACCGTGAGTGGTCAAACA | CGTCAGATGTCCGAGTAGAGGGGGAACGGCGTTGGAGTGTGACAATGGTGT |
| 11 | miR-124 | TGTCAGGCAACCGTATTCACCGTGAGTGGTGGCATT | CGTCAGATGTCCGAGTAGAGGGGGAACGGCGTAAGGCACGCGGTGAAT |
| 12 | miR-141 | TGTCAGGCAACCGTATTCACCGTGAGTGGTCCATCT | CGTCAGATGTCCGAGTAGAGGGGGAACGGCGTAACACTGTCTGGTAAAG |
| 13 | miR-145 | TGTCAGGCAACCGTATTCACCGTGAGTGGTAAGGGA | CGTCAGATGTCCGAGTAGAGGGGGAACGGCGGTCCAGTTTTCCCAGGAA |
| 14 | miR-146a | TGTCAGGCAACCGTATTCACCGTGAGTGGTAACCCA | CGTCAGATGTCCGAGTAGAGGGGGAACGGCGTGAGAACTGAATTCCA |
| 15 | miR-146b | TGTCAGGCAACCGTATTCACCGTGAGTGGTAGCCTA | CGTCAGATGTCCGAGTAGAGGGGGAACGGCGTGAGAACTGAATTCCATA |
| 16 | miR-155 | TGTCAGGCAACCGTATTCACCGTGAGTGGTACCCCT | CGTCAGATGTCCGAGTAGAGGGGGAACGGCGTTAATGCTAATCGTGATAGG |
| 17 | miR-182 | TGTCAGGCAACCGTATTCACCGTGAGTGGTAGTGTG | CGTCAGATGTCCGAGTAGAGGGGGAACGGCGTTTTGGCAATGGTAGAACTCAC |
| 18 | miR-191 | TGTCAGGCAACCGTATTCACCGTGAGTGGTAGCTGC | CGTCAGATGTCCGAGTAGAGGGGGAACGGCGCAACGGAATCCCAAAA |
| 19 | miR-203 | TGTCAGGCAACCGTATTCACCGTGAGTGGTCTAGTG | CGTCAGATGTCCGAGTAGAGGGGGAACGGCGTGTGAAATGTTTAGGACCAC |
| 20 | miR-214 | TGTCAGGCAACCGTATTCACCGTGAGTGGTACTGCC | CGTCAGATGTCCGAGTAGAGGGGGAACGGCGACAGCAGGCACAGACAGG |
| 21 | *RNU6-2* | *TGTCAGGCAACCGTATTCACCAAAAATAT* | *CGTCAGATGTCCGAGTAGAGGCTGCGCGCTAAGGATGACACG* |
| 22 | *miR-326* | *TGTCAGGCAACCGTATTCACCGTGAGTGGTCTGGAG* | *CGTCAGATGTCCGAGTAGAGGGGGAACGGCGCCTCTGGGCCCTTCCTC* |
