# Supplementary material for: Deregulation of miR‐27a may contribute to canine fibroblast activation after coculture with a mast cell tumour cell line
Source: FEBS Open Bio. 2020 Apr 1;10(5):802–16. doi: 10.1002/2211-5463.12831 (PMC7193169; doi:10.1002/2211-5463.12831)
Supplement: Supplementary file 3 — Table S3. Primer sequences of selected mRNAs. Genes for normalisation are presented in Italic. [file FEB4-10-802-s011.docx]

**Table S3. Primer sequences of selected mRNAs.** Genes for normalisation are presented in Italic.

| Nr | Gene Name | Forward Primer (5'->3') | Reverse Primer (5'->3') | Product size |
| --- | --- | --- | --- | --- |
| 1 | ACTA2 | GATCAAGATCATTGCCCCTCCG | AGACACAGAGCAAGGAAGCG | 173 |
| 2 | ASPN | TCTTACAAGTCGTAGGCTGGG | TTGTCATCATCAGGGTCGCC | 175 |
| 3 | ATM | TCTCAGGATAACCCGCAGGA | GCAGCTTCCAACAGCCTCTA | 120 |
| 4 | CBLB | CAGTCACAGGACAGACGAAATCT | TATCTCCCTGGAACTGCCCAT | 103 |
| 5 | CCDC6 | AGGCCTATATCACCCGGTCT | GAAGGAAGCCTTTGGCGTTG | 196 |
| 6 | CCND2 | TACCTTCCCCAGTGCTCCTAC | TTCCTCACAGACCTCCAGCATC | 99 |
| 7 | CCNG1 | TTGGCTTTGACACGGAGACA | TAGAAGCAGCTCAGCCCAAC | 112 |
| 8 | CD44 | ACCCCCATTACCATTCCAACC | TTCGCGGGTCCAGTTTTCTC | 177 |
| 9 | CDK6 | TGGCTCTTACTTCAGTGGTCG | CCAACACTCCAGAGGTCCAC | 97 |
| 10 | COL3A1 | GTGGCTTCCAGTTTGGCTAC | TGATCCATGTACGCGATGCT | 142 |
| 11 | CREB3L2 | CTAACCGCGGAGGCATCTC | AGGAGTTCTGAGAAGTGCGTG | 175 |
| 12 | DPT | AGAAAGAAGGCTCGGACAGAC | TAAAACTGCCACTCCCGATCC | 199 |
| 13 | EGFR | ATCGACCGAAATGGGACCTG | TGTTGTCCTCCGTCAAGGTG | 91 |
| 14 | EGLN1 | GACTGGGATGCCAAGGTAAGT | TCGTGCTCTCTCATCTGCATC | 198 |
| 15 | FAP | CGAGGAACAGCTTACCAAGG | AGACCAGTTCCTGAAGCAAGG | 142 |
| 16 | FGF11 | CCAGGCAAATCCTGACGGAA | GCTGTGAAATGTGGCGAGC | 174 |
| 17 | FN1 | GGACTCCATTCCAAGAAAGCTC | GGTCTTTCAGTGCCTCGACT | 165 |
| 18 | FOXO1 | ATTGAGCGCTTGGACTGTGA | GTGTAGCCTGTCCACTAACTCA | 175 |
| 19 | FOXO3 | GGGCAAAGCAGACCCTCAAA | TGGTGCGGGATTCACAAAGG | 119 |
| 20 | GAPDH | CTGAACGGGAAGCTCACTGG | TCCGATGCCTGCTTCACTAC | 131 |
| 21 | IL6 | GTGTGAAGACAGCAAAGAGGC | AGGTCTCCTGATTGAACCCAGA | 101 |
| 22 | JAK2 | ATGTCTGGAGCTTTGGAGTGG | ATCTGGGCATCCATCTGGTC | 182 |
| 23 | KRAS | AGTACATGAGGACTGGGGAGG | CTTGCTAAGTCCTGAGCCTGTT | 196 |
| 24 | LGALS3 | CCCAGTGCAAACAGACTTGC | GAATGGAAAAGCTGCCTGCC | 159 |
| 25 | LMNA | TACAGTGAGGAGCTGCGTGA | TCCAGCTTGGCAGAGTAGGT | 185 |
| 26 | MAPK1 | TCCCCATCACAGGAAGACCT | AATCCAGAGCTTTCGAGTCAGC | 130 |
| 27 | MDM4 | TCCGTGAAAGATCCAAGCCC | GAGAGTCTGAGCAGCATCTGT | 90 |
| 28 | PLCB1 | GGAACCCGTGTGGATTCATCT | TTGCATAGCCAGGTCCACAG | 102 |
| 29 | RAPGEF4 | TCGCCAGGGTGATATTGGAAC | GATGGTCACTGCATCCTGGT | 100 |
| 30 | ROCK2 | CCTGGGTGGTTCAGCTCTTT | AAGCCCATGGAGTGTATGGC | 181 |
| 31 | SESN2 | TGAACAACTCTGGGGGCTTT | TCAAAGCGGCTCTCCATCTC | 127 |
| 32 | SMAD2 | GGGAATCGAGCCACAGAGTAAT | ACTGGCTGCAAATCCAAACTATG | 174 |
| 33 | SMAD9 | ACACATAGGAAAGGGTGTGCAT | TTCCGGCTCTGCACAAAGAT | 93 |
| 34 | SMARCAD1 | GATGCCACAGAGTAGGCCAG | GTCCCTTCGTCACCTTCATCT | 148 |
| 35 | SOS2 | TTGGCAGAGGAGCAAGCATT | GGCGTGGAAGGACATAACGA | 169 |
| 36 | STC1 | CATTCGGAGGTGCTCCACTT | TCACATTCCAGGAGGCTTCG | 183 |
| 37 | S100A4 | CTCTTCTCCTTCTTGGTCTGGTC | CTTCATCCGTCCTTTTCCCCA | 192 |
| 38 | TGFB1 | CATTAACGGGTTCAGTTCCAGC | TCAATGTAGAGCTGCCGGAC | 198 |
| 39 | TRAF6 | GCTCATCAGAGAACAGATGCCT | ATTCTCCTGTAGGTGGCGTG | 133 |
| 40 | VCAM1 | GGTGGACCTCTACTCATTCCC | TTAGGAACCTCGCAGCTCAC | 96 |
| 41 | *HPRT1* | *CCCAGCGTCGTGATTAGTGAT* | *TTGAGCACACAGAGGGCTAC* | *191* |
| 42 | *RPS19* | *AAACCCCGTTGCTCCCTTTC* | *TCCCAGACTTTTTGAGGAAGGC* | *127* |
