# Supplementary figures and images for: Deregulation of miR‐27a may contribute to canine fibroblast activation after coculture with a mast cell tumour cell line
Source: FEBS Open Bio. 2020 Apr 1;10(5):802–16. doi: 10.1002/2211-5463.12831 (PMC7193169; doi:10.1002/2211-5463.12831)

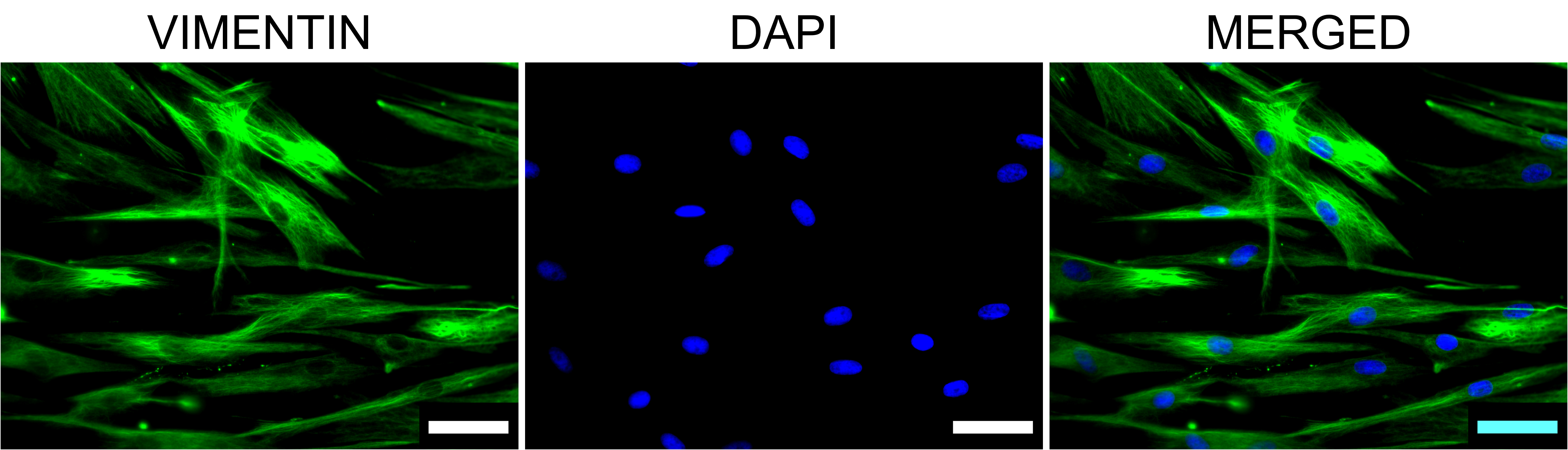

Supplement: Supplementary file 4 — Fig. S1. Immunofluorescence staining of vimentin in canine primary fibroblasts. IF representative images represent at least two biological replicates. Scale bars represent 50 μm. [file FEB4-10-802-s001.tif]

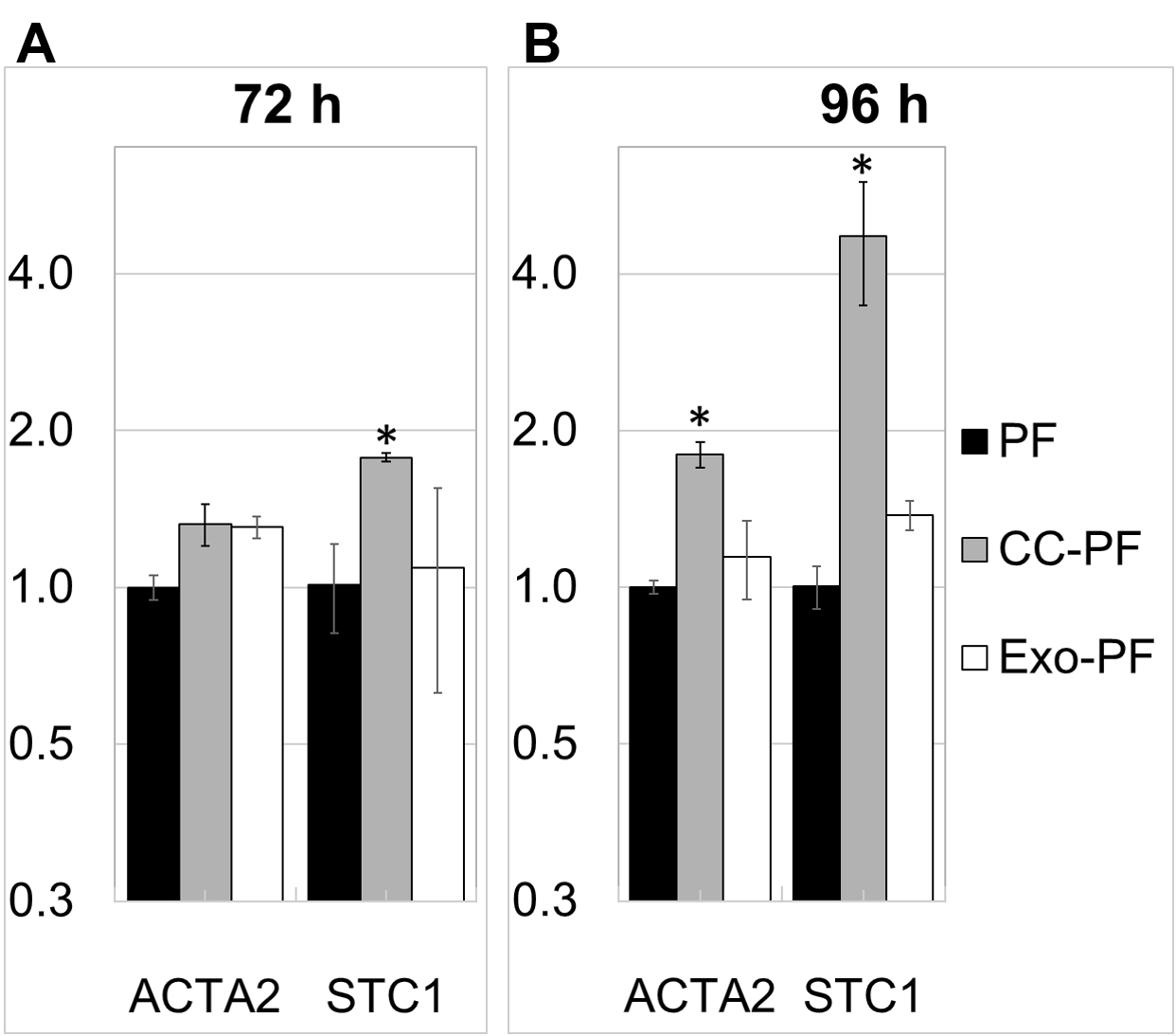

Supplement: Supplementary file 5 — Fig. S2. Relative expression of mRNA levels for CAF markers ACTA2 and STC1 in canine primary fibroblasts compared with PF control group. Expression was evaluated in PF, CC‐PF and Exo‐PF groups after (A) 72 h and (B) 96 h. Results were normalised to HPRT1 and RPS19 and analysed using of the 2‐ΔΔCT method. Datasets are expressed as means of three biological samples and duplicate measurements ± SD, analysed with a two‐tailed Student’s t‐test. Asterisks represent a statistical significance compared with the control group FB (*P =< 0.05). [file FEB4-10-802-s002.tif]

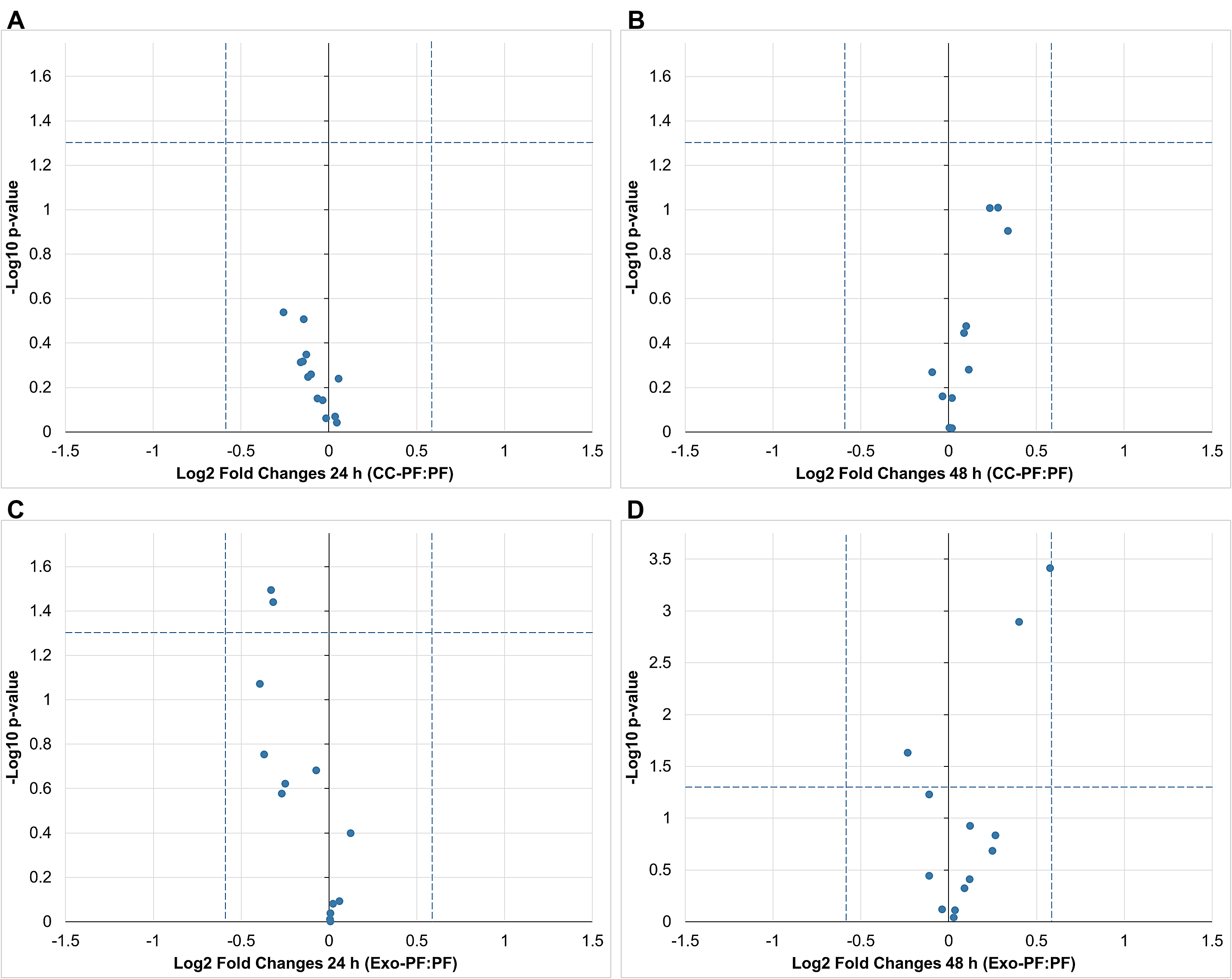

Supplement: Supplementary file 6 — Fig. S3. Relative expression of miRNAs in canine primary fibroblasts compared with PF control group. (A) 24 h and (B) 48 h after coculture with C2 cells: CC‐PF group. (C) 24 h and (D) 48 h after culture with C2‐derived exosomes: Exo‐PF group. No significantly regulated miRNAs were observed. Results were normalised to RNU6‐2 and miR‐326 and analysed using the 2‐ΔΔCT method. Datasets are expressed as means of three biological samples and triplicate measurements ± SD, analysed with a two‐tailed Student’s t‐test and transformed into log2 vs. –log10 P‐value. No statistical significance was found compared with the control group FB (P < 0.05). [file FEB4-10-802-s003.tif]

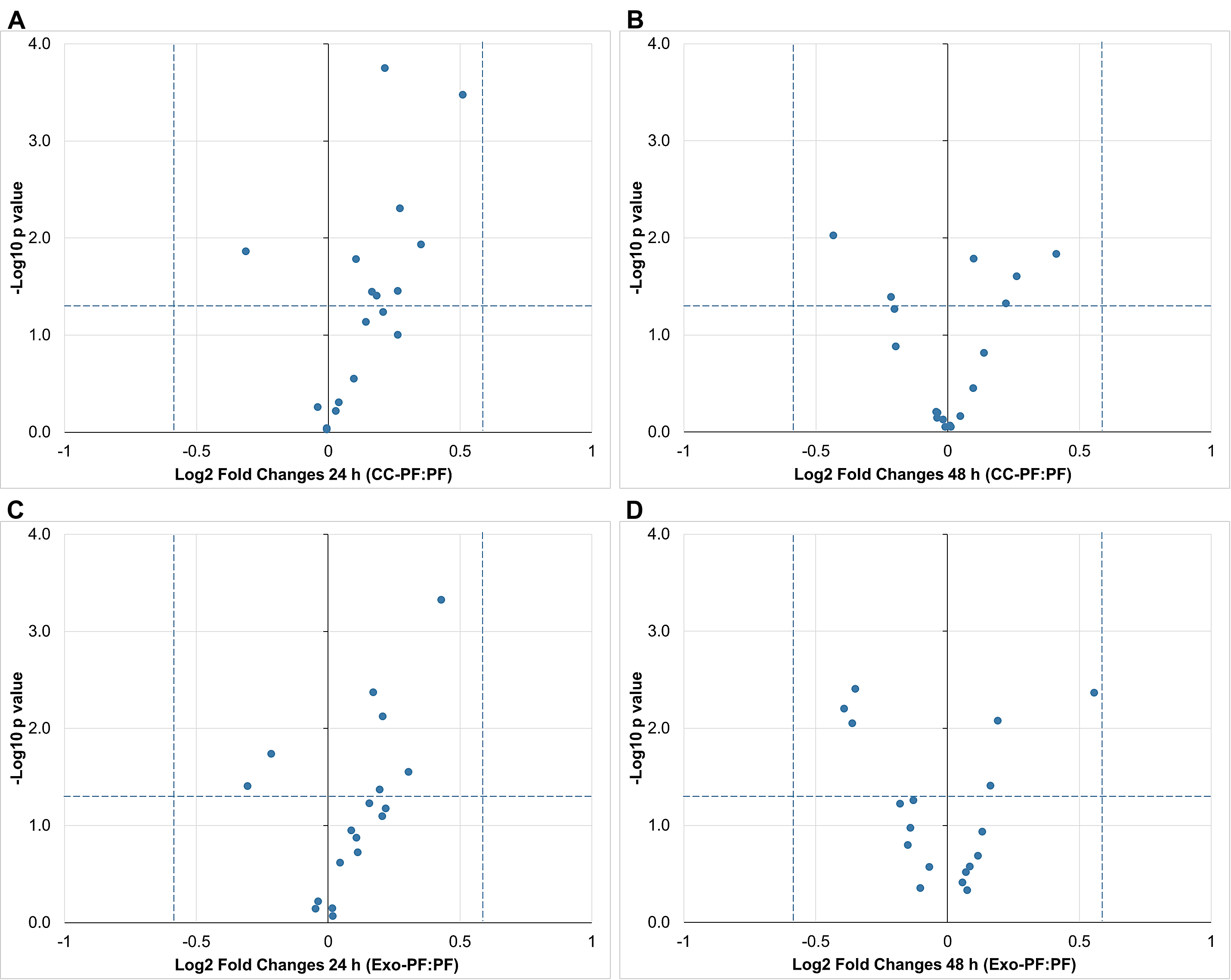

Supplement: Supplementary file 7 — Fig. S4. Relative expression of predicted target mRNAs in canine primary fibroblasts compared with PF control group. (A) 24 h and (B) 48 h after coculture with C2 cells: CC‐PF group. (C) 24 h and (D) 48 h after culture with C2‐derived exosomes: Exo‐PF group. No significantly regulated mRNAs were observed. Results were normalised to HPRT1 and RPS19 and analysed using of the 2‐ΔΔCT method. Datasets are expressed as means of three biological samples and duplicate measurements ± SD, analysed with a two‐tailed Student’s t‐test and transformed into log2 vs. –log10 P‐value. No statistical significance was found compared with the control group FB (P < 0.05). [file FEB4-10-802-s004.tif]

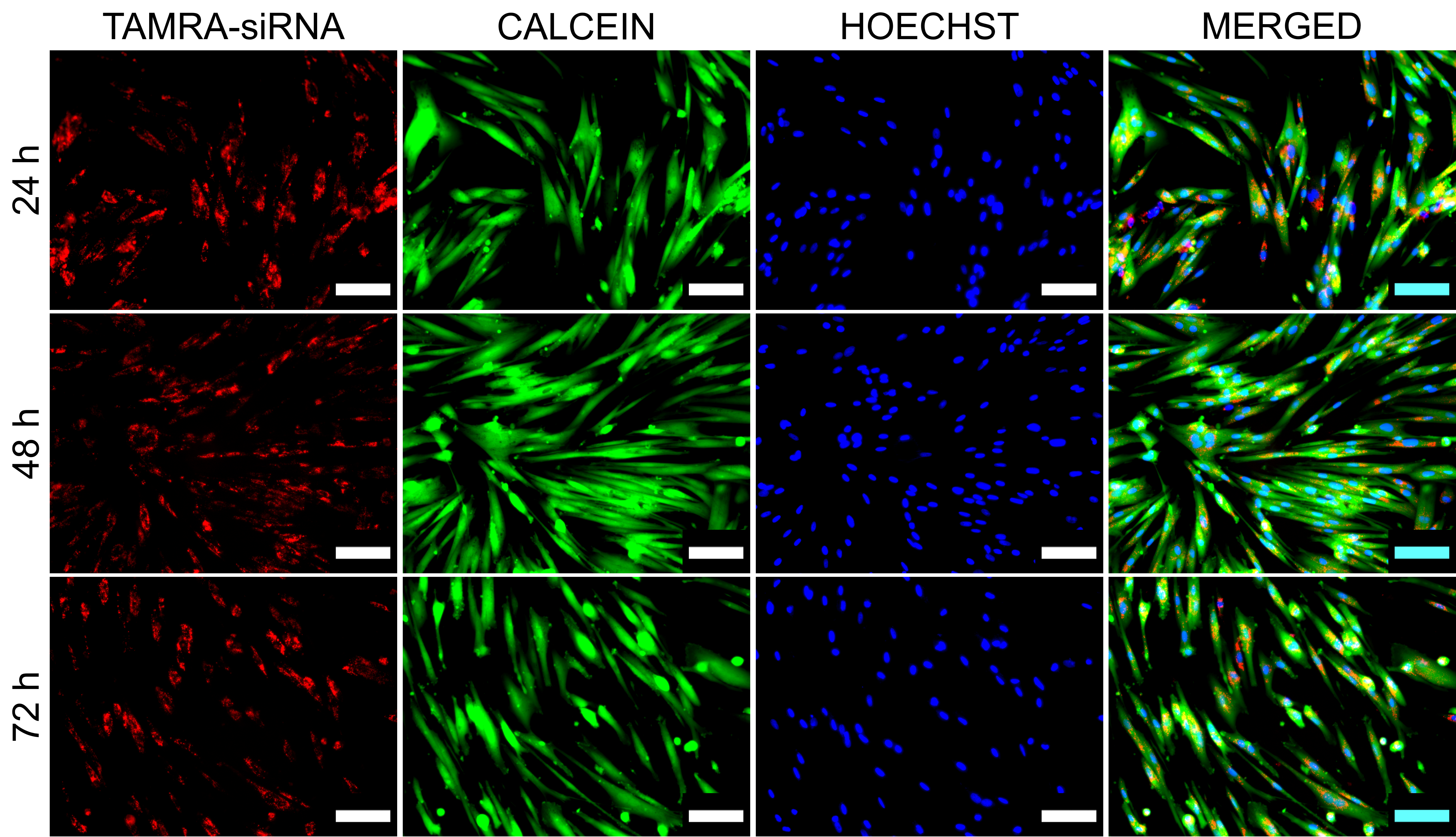

Supplement: Supplementary file 8 — Fig. S5. Representative images of transfection efficiency evaluated using a TAMRA‐labelled siRNA. At the same time, viability of primary fibroblasts transfected with fluorescent siRNA control was also evaluated through Calcein and Hoechst fluorescent staining. IF representative images using at least two biological replicates were taken under identical microscope and camera settings. Scale bars represent 100 μm. [file FEB4-10-802-s005.tif]

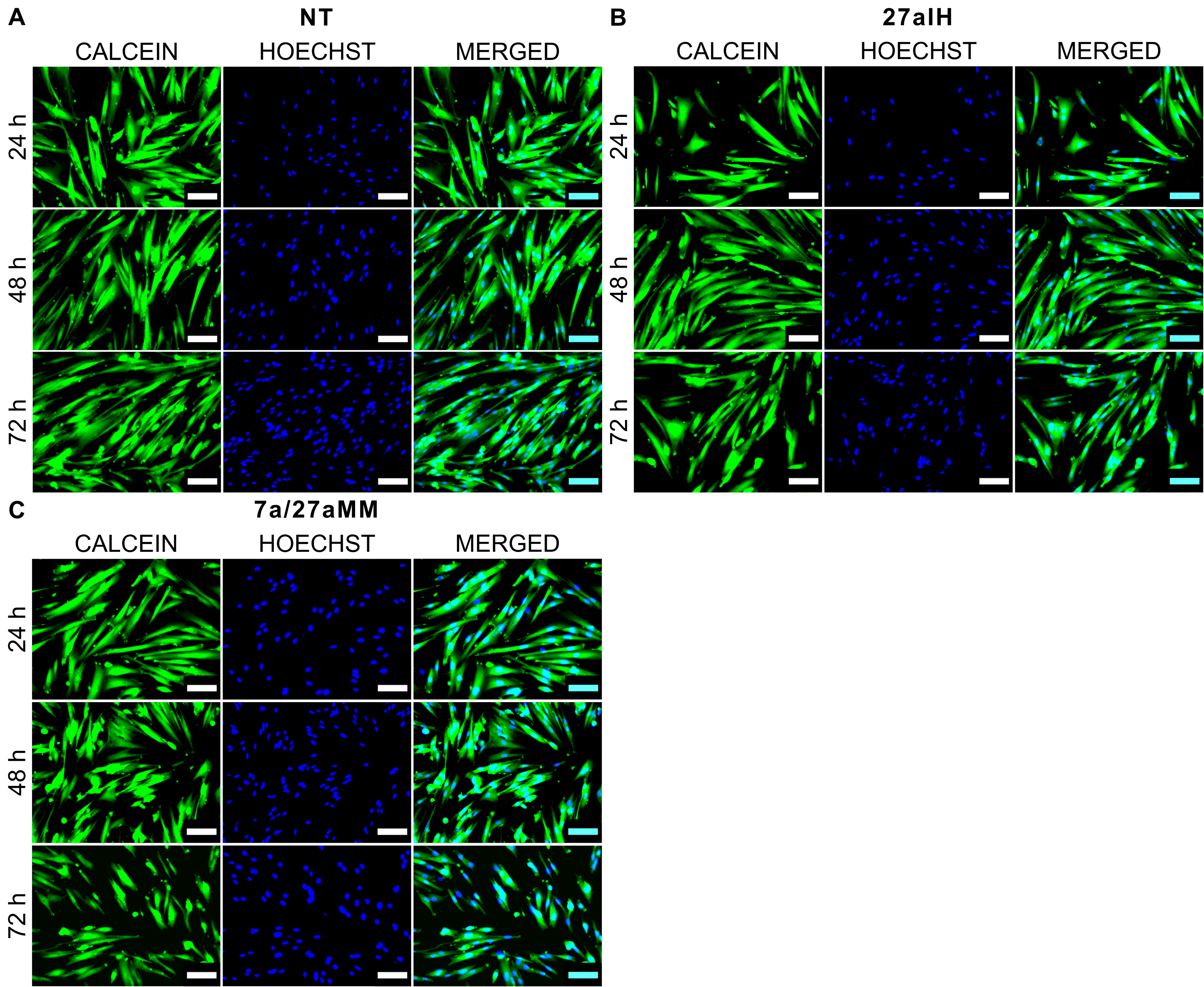

Supplement: Supplementary file 9 — Fig. S6. Representative images of cell viability in primary fibroblasts evaluated by using Calcein and Hoechst fluorescent staining. (A) Cellular viability in NT group, (B) cellular viability in 27aIH group and (C) cellular viability in 7a/27aMM group. IF representative images using at least two biological replicates were taken under identical microscope and camera settings. Scale bars represent 100 μm. [file FEB4-10-802-s006.tif]

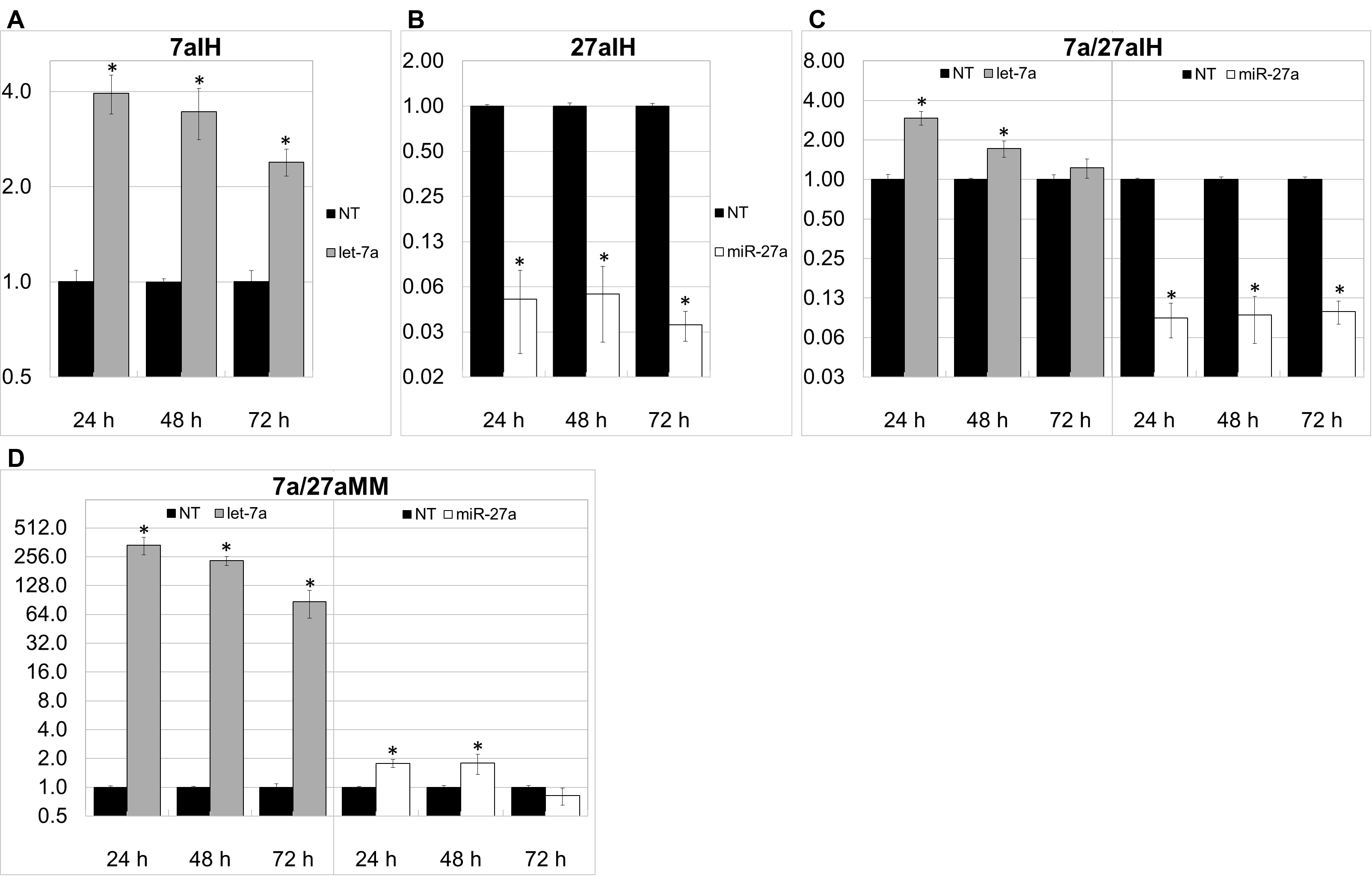

Supplement: Supplementary file 10 — Fig. S7. Relative expression of transfected miRNAs in primary fibroblasts compared with NT group. (A) 7aIH, (B) 27aIH, (C) 7a/27aIH and (D) 7a/27aMM groups. Results were normalised to RNU6‐2 and miR‐326 and analysed using the 2‐ΔΔCT method. Datasets are expressed as means of three biological samples and triplicate measurements ± SD, and analysed with a two‐tailed Student’s t‐test. Asterisks represent a statistical significance compared with the control group NT (*=P < 0.05). [file FEB4-10-802-s007.tif]

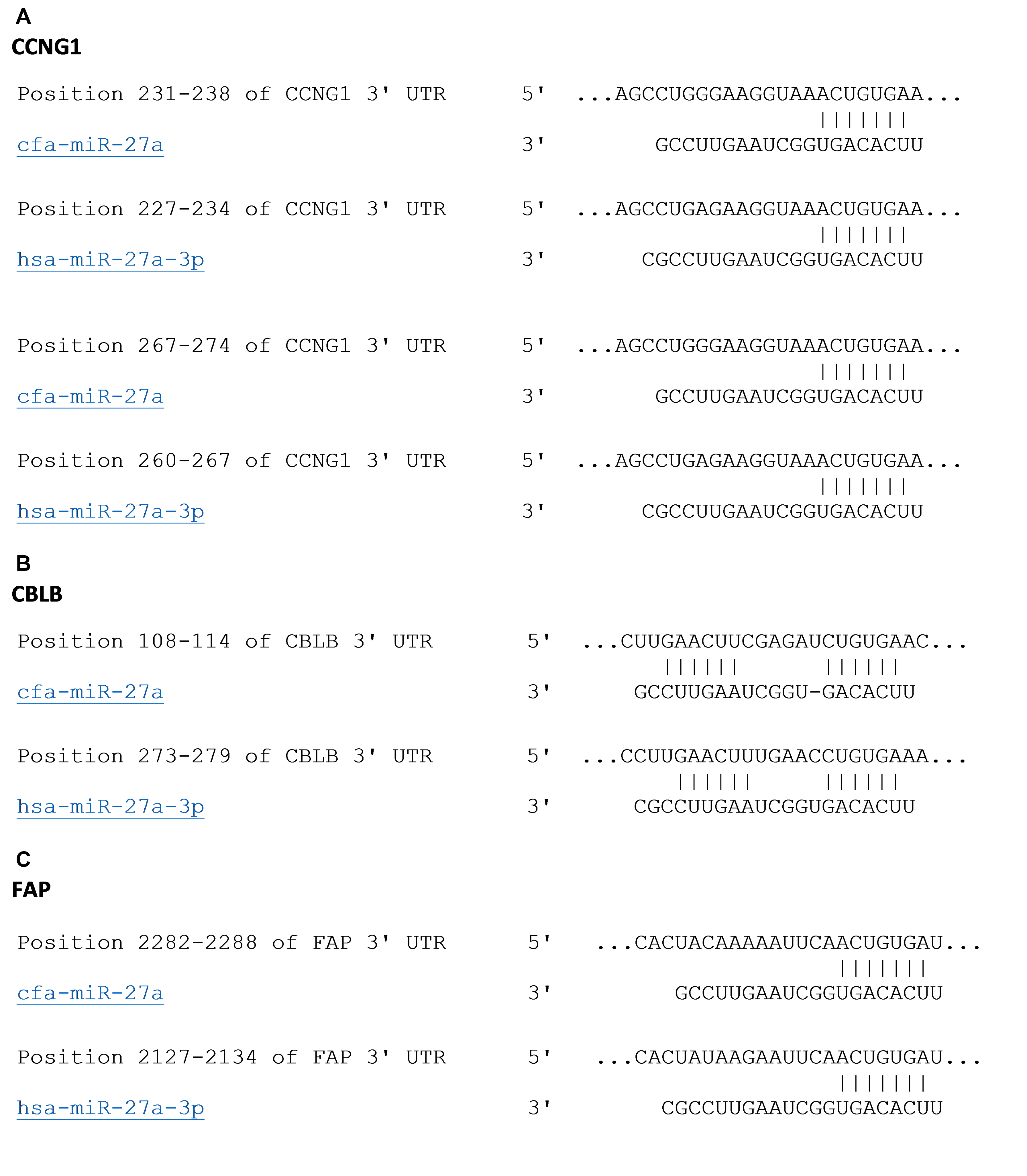

Supplement: Supplementary file 11 — Fig. S8. Canine predicted binding sites in the 3’ UTR region of regulated genes after miR‐27a transfection, compared to human. Predicted interaction of each gene’s target region (top) with miR‐27a (bottom). (A) CCNG1 sequence has in both species 2 miR‐27a binding sites in different positions due to nucleotide repetition. (B) CBLB and (C) FAP sequences have in both species a single miR‐27a binding site. Pairing between genes and miR‐27a was performed using the online resources TargetScan and RNAhybrid. [file FEB4-10-802-s008.tif]
